# Cortisol-resistant CAR-NK cells overcome steroid-induced immunosuppression in lung cancer

**DOI:** 10.1038/s41392-026-02638-z

**Published:** 2026-04-09

**Authors:** Soura Chakraborty, Jhuma Pramanik, Gustavo Alviter-Raymundo, Christopher J. Ward, Sanu K. Shaji, Yumi Yamashita-Kanemaru, Fatma Abo Zakaib Ali, Debasis Banik, Ziwei Zhang, Clara Veiga-Villauriz, Natalie Z. M. Homer, Joanna Simpson, Sofia Laforest, Shanlin Tong, Qiuchen Zhao, James Roy, Muhammad Iqbal, Andrew Conway Morris, Michael A. Chapman, Rahul Roychoudhuri, Hosni Hussein, David Klenerman, Kourosh Saeb-Parsy, Bidesh Mahata

**Affiliations:** 1 Department of Pathology, University of Cambridge, Cambridge, UK; 2 Department of Surgery, University of Cambridge and NIHR Cambridge Biomedical Research Centre, Cambridge, UK; 3 Department of Pathology and Clinical Pathology, Faculty of Veterinary Medicine, Sohag University, Sohag, Egypt; 4 Department of Anatomy and Comparative Pathological Anatomy and Toxicology, University of Córdoba, Córdoba, Spain; 5 Yusuf Hamied Department of Chemistry, University of Cambridge, Cambridge, UK; 6 Mass Spectrometry Core, Edinburgh Clinical Research Facility, Institute for Neuroscience and Cardiovascular Research, Queens Medical Research Institute, University of Edinburgh, Edinburgh, UK; 7 Department of Haematology, University of Cambridge, Puddicombe Way, Cambridge, UK; 8 Division of Perioperative, Acute, Critical Care and Emergency Medicine, Department of Medicine, University of Cambridge, Addenbrooke’s Hospital, Cambridge, UK; 9 Department of Microbiology, Faculty of Science, Al-Azhar University, Assiut, Egypt

**Keywords:** Tumour immunology, Lung cancer, Immunotherapy

## Abstract

Tumors foster an immunosuppressive microenvironment to evade the antitumor immune response. However, the influence of intratumoral immunosuppressive steroids on tumor-infiltrating natural killer (NK) cells and their implications for effective immunotherapy has remained largely unexplored. Here, we report that the functional enrichment of glucocorticoid cortisol signaling in the lung tumor microenvironment (TME) impairs NK cell anti-tumor cytotoxicity and exacerbates hypoxic stress. Cancer-associated fibroblasts (CAFs) and macrophages convert inactive cortisone to active cortisol, while T cells, fibroblasts, myeloid cells, macrophages, and cancer cells contribute to de novo steroid biosynthesis, collectively establishing a steroid-rich niche. Pharmacological inhibition of the glucocorticoid receptor (GR) in vivo alleviates cortisol-mediated immune suppression, resulting in reduced tumor growth and enhanced cytotoxicity of tumor-infiltrating NK cells. To overcome the cortisol-induced dysfunction of solid tumor targeting immunotherapy, we engineered chimeric antigen receptor (CAR) -NK cells specific to the Carcinoembryonic antigen-related cell adhesion molecule 5 (CEACAM5) (highly expressed in lung tumors) and rendered them cortisol-resistant by genetic deletion of the cortisol receptor gene *NR3C1*. In cortisol-rich niches, cortisol-resistant CAR-NK cells sustained antitumor cytotoxicity. Mechanistically, *NR3C1* deletion relieved cortisol-mediated suppression of PI3K-AKT-NF-κB signaling, restored anti-tumor activity, and markedly reduced hypoxic stress. In lung metastasis models, cortisol-resistant CAR-NK cells achieved superior tumor control and significantly reduced tumor burden compared with conventional CAR-NK cells. Together, these findings identify local cortisol signaling as a critical barrier to solid tumor immunotherapy and establish cortisol-resistant CAR-NK cells as a promising strategy for targeting steroidogenic solid tumors, which can be combined with therapeutic glucocorticoids.

## Introduction

The tumor microenvironment (TME) establishes an immunosuppressive microenvironment to evade immune surveillance.^1^ Within the TME, hypoxia, low pH, and accumulation of immunosuppressive metabolites, including lactate, oxidized lipids, and adenosine, hamper the effector function of cytotoxic T lymphocytes (CTLs) and NK cells by reducing cytokine production and inducing an exhausted phenotype.^2,3^ Although steroids are well-known for their systemic immunosuppressive effects, recent studies indicate that tumors may exploit de novo steroid biosynthesis as an additional strategy to evade anti-tumor immunity.^4–7^ Glucocorticoids in particular have been reported to promote metastasis.^8^ However, the effects of local glucocorticoids on evading anti-tumor immunity by modulating NK cell function remain largely unexplored. Immunosuppressive steroid accumulation within the TME may result from local steroid biosynthesis, steroid metabolism, stress-induced systemic elevation, or administration of steroid medications as part of palliative care in cancer patients.^9–11^ Consistent with this emerging view, local steroid production or regeneration has been described across diverse solid malignancies, including colorectal cancer, breast cancer, pancreatic ductal adenocarcinoma, and melanoma, raising the possibility that intratumoral glucocorticoid signaling constitutes an underappreciated axis of immune suppression in solid tumors.^9,12^

Immunotherapy has revolutionized cancer treatment; however, durable responses in solid tumors remain limited due to profound immunosuppression within the TME. NK cells are key cytotoxic effectors within the innate immune system, directly targeting tumor cells and facilitating the recruitment of adaptive immune cells such as T cells and dendritic cells through the cytokines and chemokines production.^13^ NK cells, with their broad tumor recognition and potent anti-tumor activity, are promising candidates for chimeric antigen receptor (CAR) development in cancer immunotherapy. Several CAR-NK cell therapies are in clinical trials, showing promising treatment strategies.^14^ However, their efficacy against solid tumors is frequently limited by poor trafficking and infiltration as well as immunosuppressive cues within the TME. Exposure to glucocorticoids can significantly alter NK cell function, reducing cytotoxicity and impairing cytokine production. Glucocorticoids can further modulate the expression of activating and inhibitory receptors on NK cells, thereby contributing to immune evasion and tumor progression.^15,16^ Lung cancer is an aggressive and widespread disease globally.^17^ In non-small cell lung cancer (NSCLC) and urothelial carcinoma, therapeutic glucocorticoids are associated with poor survival in cancer patients.^18^ Several NSCLC and small cell lung carcinoma (SCLC) cell lines express key steroidogenic enzymes and produce bioactive glucocorticoids under steady-state conditions.^19^ Although systemic steroid immunosuppression is well recognized, and glucocorticoids are routinely administered to manage inflammation and treatment-related toxicities, the abundance of cortisol in the lung TME and its impact on lung tumor-infiltrating NK cells remain unexplored.

Using quantitative mass spectrometry-based steroid profiling combined with single-cell transcriptomic analyses, we defined the steroid landscape of lung cancer and examined its influence on tumor-infiltrating NK-cell cytotoxicity. We mapped *CYP11A1*-expressing steroidogenic cell types and also identified steroid-recycling cells that regenerate active cortisol via HSD11B1-mediated cortisone-to-cortisol conversion within the lung TME. In murine lung tumor models, we characterized cortisol-driven dysfunction of lung tumor-infiltrating NK cells and found that restoring effector function required pharmacological inhibition of glucocorticoid receptor (GR) signaling. In parallel, we delineated the mechanisms underpinning cortisol-induced NK-cell suppression and translated these insights into a therapeutic strategy by engineering cortisol-resistant, CEACAM5-specific CAR-NK cells.

In this study, we identify cortisol as a dominant steroid enriched within the lung TME and show that glucocorticoid signaling in tumor-infiltrating NK cells is associated with reduced cytotoxicity, increased dysfunction, and an amplified hypoxic stress signature. We define cancer-associated fibroblasts (CAFs) and macrophages as key hubs for cortisol regeneration, the conversion of inactive cortisone to active cortisol, while T cells, myeloid cells, fibroblasts, macrophages, and cancer cells contribute to de novo steroid biosynthesis, together establishing a cortisol-rich immunosuppressive microenvironment. Pharmacological inhibition of GR signaling in vivo reinvigorates intratumoral NK-cell activation and cytotoxicity and reduces tumor progression. To translate these findings, we engineered CEACAM5-specific CAR-NK cells and rendered them cortisol-resistant by CRISPR-mediated *NR3C1* deletion; these cells preserve PI3K-AKT-NF-κB signaling and effector cytokine production in cortisol-rich niches and exhibit attenuated hypoxic stress signatures. In a steroid-rich lung metastasis model, cortisol-resistant CAR-NK cells achieve superior tumor control and reduced metastatic burden compared with conventional CAR-NK cells. Collectively, our work defines intratumoral cortisol signaling as a key immunosuppressive axis in lung cancer and highlights cell-intrinsic glucocorticoid resistance as a tractable strategy to enhance NK-cell-based immunotherapy in cortisol-rich solid tumors.

## Results

### Glucocorticoid signaling within the lung TME suppresses the function of tumor-infiltrating NK cells

**Fig. 1:**
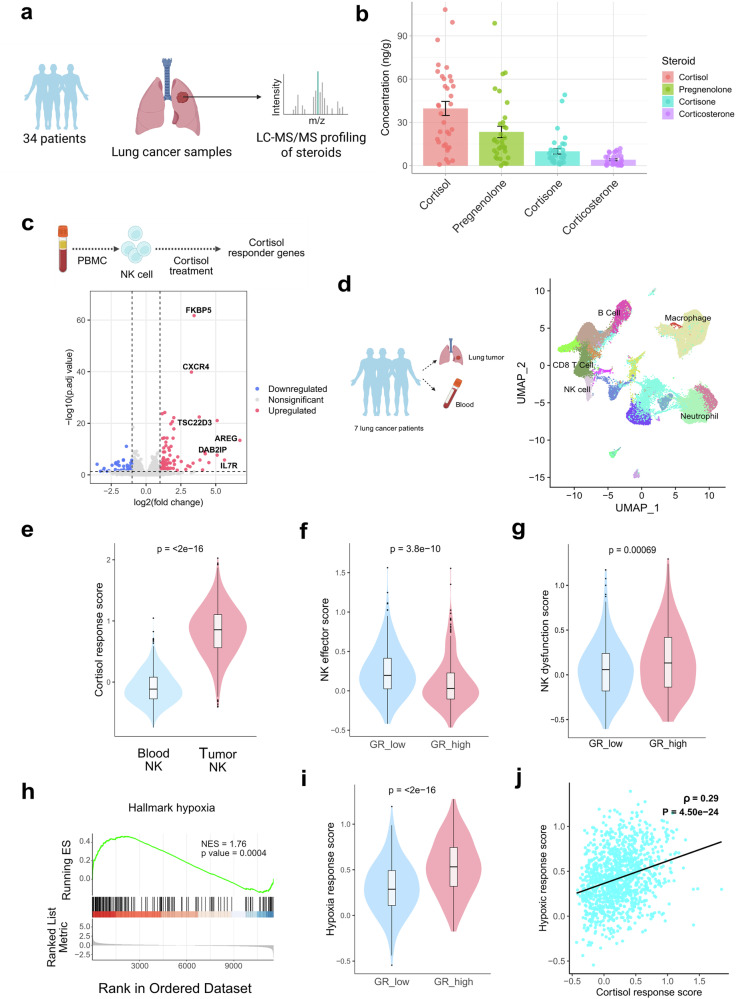
Effects of glucocorticoids on lung tumor-infiltrating NK cells. **a** Schematic representation showing the steroid profiling from 34 lung cancer patients using LC-MS/MS. The illustration was created using BioRender. **b** Quantitative detection of steroids from 34 patient samples by LC-MS/MS after organic solvent extraction. Bars represent the mean concentration ± SEM of each steroid in ng/g of lung tumor tissue. **c** Identification of cortisol (one type of glucocorticoid) responder genes in human primary NK cells. Cortisol-responder genes have been identified following a transcriptomics study of human primary NK cells treated with the glucocorticoid cortisol (also known as hydrocortisone). Volcano plot showing differentially expressed genes in human primary NK cells after 6 hours of treatment with 1 μM cortisol. The significantly upregulated genes are shown in red, and the significantly downregulated genes in blue. Several canonical glucocorticoid-responsive genes (*FKBP5, CXCR4, TSC22D3, AREG, DAB2IP, IL7R*) were significantly upregulated. log2-Fold change versus –log10(adjusted *p* value) has been plotted, *n* = 4 human samples in each group. **d** Uniform manifold approximation and projection (UMAP) visualization of single-cell transcriptomes from blood and lung tumor samples of seven patients (GSE127465) depicting major immune populations, including NK cells, CD8⁺ T cells, macrophages, neutrophils, and B cells. The illustration was created using BioRender. **e** Violin plot showing significantly higher cortisol-response scores in tumor-infiltrating NK cells compared with peripheral blood NK cells (*p* < 2 × 10⁻^16^, Wilcoxon test), indicating prominent glucocorticoid response in the tumor microenvironment. **f** Violin plot showing NK effector-function scores in glucocorticoid receptor (GR)-low versus GR-high NK cells in lung TME, demonstrating that glucocorticoid response downregulates the effector function in NK cells. (*p* = 3.8 × 10^−10^, Wilcoxon test). **g** Violin plot showing NK dysfunction scores in glucocorticoid receptor (GR)-low versus GR-high NK cells in lung TME, demonstrating glucocorticoid response influence NK cell dysfunction. (*p* = 0.00069, Wilcoxon test). **h** Gene set enrichment analysis (GSEA) showing enrichment of the Hallmark hypoxia gene signature in human NK cells treated with cortisol (NES = 1.76, *p* = 0.0004). **i** Violin plot demonstrating elevated hypoxia-response scores in high glucocorticoid receptor expressing (GR-high) NK cells in lung TME (*p* < 2 × 10^−^^16^, Wilcoxon test). **j** Scatterplot showing a positive correlation between cortisol-response and hypoxia-response scores in lung tumor infiltrating NK cells (*ρ* = 0.29, *p* = 4.5 × 10^−24^, Spearman correlation)

To investigate the impact of steroids on lung tumor-infiltrating NK cells, we first quantified the abundance of steroid metabolites within the human lung TME (Fig. 1a). Targeted liquid chromatography-tandem mass spectrometry (LC-MS/MS) analysis of tumor samples from 34 patients showed that cortisol is the most abundant steroid in lung TME, with an average concentration of 42.47 ng/g of lung tumor tissue, followed by pregnenolone (24.7 ng/g), cortisone (9.7 ng/g), and corticosterone (4.38 ng/g) in lung tumor tissue (Fig. 1b). Cortisol (glucocorticoid) and pregnenolone (precursor of all steroids in the de novo steroid biosynthesis pathway), emerged as the predominant steroids enriched within the lung TME. In-depth steroid profiling of 20 patient samples revealed the presence of multiple steroid classes, including glucocorticoids, mineralocorticoids, androgens, estrogens, and progestogens (Supplementary Table 1) (Supplementary Fig. 1a). However, we did not find any significant alteration of cortisol level in different stages of lung cancer (Supplementary Fig. 1b).

To further examine the functional effects of glucocorticoids in lung TME, we investigated cortisol-regulated genes and pathways in human NK cells and compared their expression patterns and abundance in tumor-infiltrating NK cells using single-cell RNA sequencing (scRNA-seq) data. Transcriptomics analysis of cortisol-treated human primary NK cells identified glucocorticoid-influenced gene signatures in NK cells, which include previously documented glucocorticoid-responsive genes, including *TSC22D3, AREG, DAB2IP, FKBP5*, and *IL7R* (Fig. 1c, Supplementary Fig. 1c). Consistent findings were observed in activated human primary NK cells treated with hydrocortisone (cortisol) (Supplementary Fig. 1c). We observed the downregulation of several cortisol-influenced activation markers (*NKG7, IFNG*), adhesion markers (*ITGAL, ITGB7, SLAMF7, ICAM1*), alongside upregulation of inhibitory markers (*KLRD1, CTLA4*) of human primary NK cells (Supplementary Fig. 1c, Supplementary Tables 2 and 3). Single-cell RNA sequencing (scRNA-seq) analysis of non-small cell lung cancer (NSCLC) tumors and peripheral blood samples from seven patients (GSE127465) revealed a markedly elevated cortisol-responsive transcriptional program within the TME (Fig. 1d, e).^20^ Notably, tumor-infiltrating NK cells exhibiting higher GR expression displayed reduced effector signatures (expression of NK cell effector genes including *PRF1, GZMB, IFNG, TNF, NKG7, KLRK1, DNAM1, EOMES, STAT5A, STAT5B*) compared with their low-GR expressing counterparts, indicating steroid-mediated suppression of NK-cell function in lung TME (Fig. 1f). We also observed higher GR-expressing tumor-infiltrating NK cells are showing higher dysfunction (expression of NK cell dysfunction genes including *NR4A1, NR4A2, NR4A3, SOCS1, NFATC1, CBLB*) (Fig. 1g). In-depth Gene set enrichment analysis (GSEA) analysis with hallmark pathways reveals that glucocorticoid upregulates hypoxic gene signature in NK cells beyond the direct functional inhibition of NK cells (Fig. 1h, Supplementary Fig. 1d). Analysis of scRNA-seq data revealed that among tumor-infiltrating NK cells, those with higher GR expression exhibited a pronounced hypoxic stress signature (expression of hypoxia induced genes including *HIF1A, DDIT4, PFKFB3, ALDOA, CXCR4, ETS1, ATF3, JUN, HSPA5*) compared with GR^low^ NK cells (Fig. 1i). Moreover, within lung tumor NK cells, the cortisol response score positively correlated with the hypoxic response score (Fig. 1j).

By comparing glucocorticoid responses in tumor-infiltrating and adjacent tissue-resident NK cells, we observed that tumor-infiltrating NK cells exhibited markedly stronger steroid signaling activity. Single-cell transcriptomic analysis of lung adenocarcinoma samples (GSE131907) revealed elevated expression of the GR (*NR3C1*) and its downstream target genes in tumor-infiltrating NK cells relative to their tissue-resident counterparts (Supplementary Fig. 1e). This elevated glucocorticoid responsiveness was accompanied by increased hypoxia-associated transcriptional signatures, indicating that tumor-infiltrating NK cells experience both enhanced steroid signaling and hypoxic stress within the TME (Supplementary Fig. 1e).^21,22^

Furthermore, we observed that hypoxic stress and glucocorticoid influence were not restricted to tumor-infiltrating NK cells but were evident across multiple immune populations within the lung TME, including T cells, B cells, macrophages, and dendritic cells. In a larger cohort comprising 103 patients, we observed a positive correlation between cortisol response and hypoxic response across major immune cell subsets in the TME, such as T cells, mast cells, dendritic cells, B cells, macrophages, monocytes, and NK cells in lung TME (Supplementary Fig. 2a).^23^ A similar correlation was also observed in non-immune stromal compartments, including fibroblasts and endothelial cells (Supplementary Fig. 2a). Consistently, tumor-infiltrating immune and stromal cells with higher *NR3C1* expression exhibited elevated hypoxic stress signatures, mirroring the pattern observed in NK cells (Supplementary Fig. 2b). Collectively, these findings indicate that glucocorticoid-hypoxia crosstalk extends beyond NK cells to influence the cellular landscape of the lung TME broadly. These findings suggest that elevated cortisol levels within the lung TME reprogram tumor-infiltrating NK cells, attenuating their cytotoxic potential while amplifying hypoxia-associated transcriptional programs.

### CAFs and macrophages enrich cortisol in the tumor by glucocorticoid metabolite recycling

**Fig. 2:**
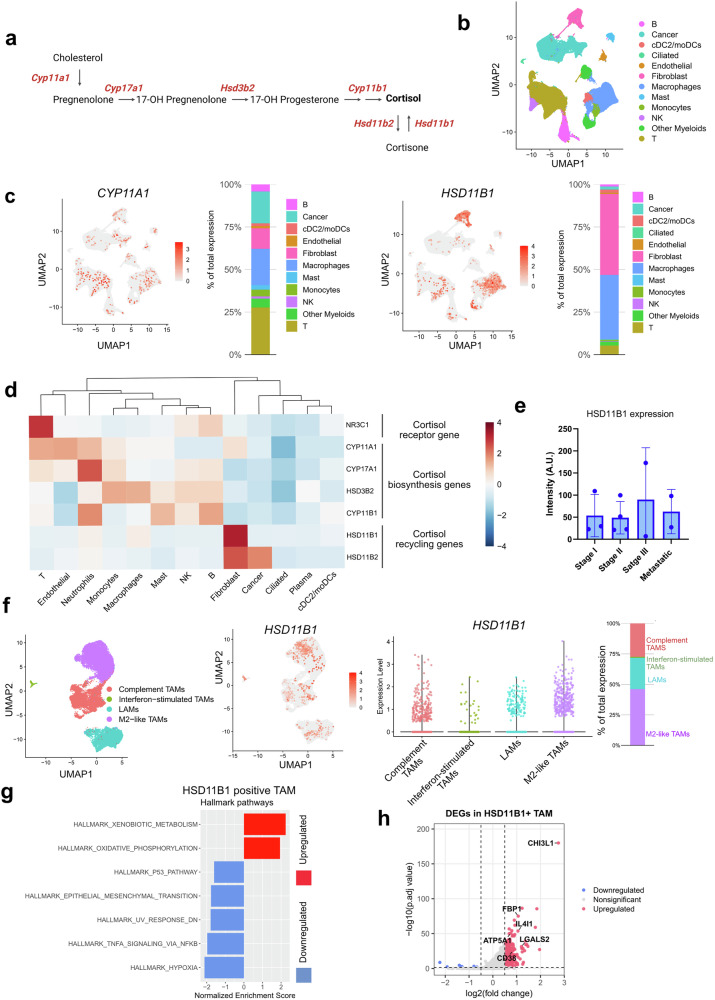
De novo steroid biosynthesis and metabolic recycling of glucocorticoid in lung TME. **a** Schematic diagram showing de novo steroid biosynthesis from cholesterol to cortisol and metabolite recycling of cortisone to cortisol. CYP11A1 is the first and key rate-limiting enzyme of de novo steroid biosynthesis from cholesterol, whereas HSD11B1 converts inactive cortisone to active cortisol, and HSD11B2 works in reverse. The illustration was created using BioRender. **b** Integrated single-cell RNA-sequencing (scRNA-seq) dataset comprising 103 lung cancer patients showing UMAP-based clustering of major cell populations, including endothelial, fibroblast, myeloid, and lymphoid subsets. **c** UMAP feature plots showing the expression of *CYP11A1* (left) and *HSD11B1* (right) across all cell types, along with corresponding bar plots denoting the relative contribution of each cell type to total gene expression. **d** Heatmap summarizing the average expression (Z-score) of steroidogenic (*CYP11A1*, *CYP17A1, HSD3B2, CYP11B1*) and cortisol-recycling (*HSD11B1*, *HSD11B2*) genes across major immune and stromal populations. Fibroblasts and macrophages displayed marked enrichment of *HSD11B1*. **e** Immunostaining (confocal microscopy) analysis of HSD11B1 expression across 11 tumor samples spanning Stage I (*n* = 3), Stage II (*n* = 4), Stage III (*n* = 2), and metastatic disease (*n* = 2). **f** UMAP visualization and violin plots highlighting macrophage heterogeneity in HSD11B1 expression across major subsets. M2-like tumor-associated macrophages (TAMs) demonstrated dominant *HSD11B1* expression. Lipid-associated macrophages are abbreviated as LAMs. **g** Hallmark pathways were significantly enriched in HSD11B1^+^ TAMs compared with HSD11B1^-^ TAMs. Positive NES values indicate upregulated pathways (red), whereas negative NES values indicate downregulated pathways (blue). **h** Volcano plot showing differentially expressed genes in HSD11B1^+^ TAMs. Significantly upregulated genes (red) include immunosuppressive and metabolic mediators such as *CHI3L1, LGALS2, IL4I1, CD38, ATP5A1*, and *FBP1* (FDR < 0.05)

Given the abundance of glucocorticoids in the lung TME and their signaling effects on lung tumor-infiltrating NK cells, we sought to identify the cells contributing to glucocorticoid enrichment in the lung TME. In the de novo steroid biosynthesis pathway, CYP11A1 (also known as P450 side chain cleavage enzyme) is responsible for converting cholesterol to pregnenolone, from where the other downstream steroids are synthesized (Fig. 2a). In the metabolic conversion of steroids, HSD11B1 (also known as hydroxysteroid 11-beta dehydrogenase 1) enzyme is responsible for converting inactive cortisone to active cortisol. The single cell transcriptomics of 224611 cells from 103 patient samples collected from different datasets (GSE148071, KU_loom, GSE153935, GSE131907, GSE136246, GSE119911, GSE127465), including NSCLC, squamous cell carcinoma, from different stages (Stage I, II, III, IV) reveals de novo steroid biosynthesis and metabolite recycling of glucocorticoids, enrich the cortisol level in lung TME.^23^ T cells, myeloid cells, fibroblasts, macrophages, and cancer cells emerge as primary sources of de novo steroid biosynthesis, with high *CYP11A1* expression (Fig. 2b, c). In contrast, CAFs and macrophages primarily convert inactive cortisone to active cortisol, with elevated *HSD11B1* expression (Fig. 2c). In the lung TME, expression of cortisol biosynthetic genes is compartmentalized within the immune cell types and stromal cells (Fig. 2d). Though it is known that GR (encoded by *NR3C1*) is ubiquitously expressed in all living cells, in our analysis we observed heightened expression in T, NK and B cells making them steroid-responder cells (Fig. 2d). However, we did not observe significant stage-specific variation for *HSD11B1* and *CYP11A1* expression in lung cancer (Fig. 2e, Supplementary Fig. 3a, b). In the TCGA LUSC cohort, higher HSD11B1 expression trended toward worse overall survival (*p* = 0.05). (Supplementary Fig. 3c).

HSD11B1, the enzyme that regenerates active cortisol from cortisone, was predominantly expressed in CAFs and tumor-associated macrophages (TAMs). Within the myeloid compartment, *HSD11B1* expression was distributed across macrophage subsets but was disproportionately enriched in M2-like TAMs (Fig. 2f). GSEA of *HSD11B1* expressing macrophages in lung cancer revealed that HSD11B1⁺ TAMs were characterized by strong induction of xenobiotic-metabolism pathways and broad repression of p53, epithelial mesenchymal transition (EMT), TNFα-NF-κB signaling, indicative of a metabolically adapted macrophage state with dampened inflammatory signaling (Fig. 2g). Transcriptional profiling of *HSD11B1* expressing tumor associated macrophages (TAMs) further showed enrichment of immunoregulatory mediators, including *CHI3L1, FBP1, LGALS2, CD38* and the potent T-cell inhibitor *IL4I1*, collectively defining a cortisol-amplifying macrophage population with pronounced immune-suppressive potential (Fig. 2h, Supplementary Table 4).

Across fibroblast subsets, *HSD11B1* expression was predominantly localized to inflammatory CAFs (iCAFs) (Supplementary Fig. 3d).^24^ Like *HSD11B1*^+^ TAMs we observed, *HSD11B1*⁺ CAFs displayed selective upregulation of xenobiotic-metabolism signaling and coordinated downregulation of cell-cycle, cytoskeletal, and epithelial-mesenchymal transition (EMT) program, consistent with a non-proliferative, metabolically rewired phenotype (Supplementary Fig. 3e). These fibroblasts expressed immune-suppressive and tumor-supportive mediators, including *SEPP1, SOD3, MGP, EMILIN1, CXCL12, TGFB1, SERPINF1*, and *IL15RA*, alongside reduced NF-κB activity, supporting ECM stiffening and impaired cytotoxic-lymphocyte function (Supplementary Fig. 3f, Supplementary Table 4). *HSD11B1*⁺ iCAFs demonstrated active steroid recycling through elevated HSD11B1 and CYP enzymes, secretion of IL6, IL33, CXCL12, TNFSF14, and PTGER2/4, and suppression of interferon program (Supplementary Fig. 3g). Tumors enriched for *HSD11B1*⁺ iCAFs exhibited increased exhaustion of CD8⁺ T and NK cells, implicating this stromal population in broad immunosuppressive reprogramming of the lung TME (Supplementary Fig. 3h).

### Inhibition of the GR enhances the cytotoxicity of lung tumor-infiltrating NK cells

**Fig. 3:**
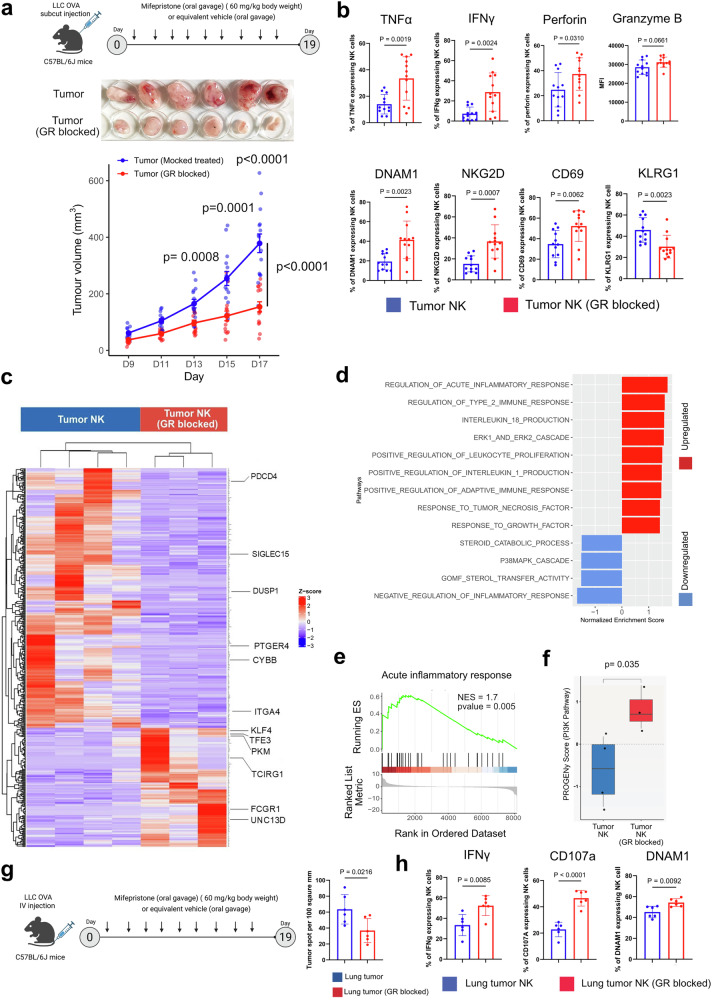
Inhibition of the glucocorticoid receptor enhances the cytotoxicity of lung tumor-infiltrating NK cells. **a** Top panel: the schematic representation of the study we performed in C57BL/6 J mice. One million LLC-OVA cells were injected subcutaneously into each mouse. From there, 15 mice were treated with the glucocorticoid receptor inhibitor mifepristone (60 mg/kg body weight) every other day, and another 15 mice were treated with vehicle (olive oil) through oral gavage for 18 days. On the 19th day^,^ mice were sacrificed. The illustration was created using BioRender. Middle panel: representative photograph of the tumors at the end-point of the study at Day 19. Bottom panel: the plot representing the subcutaneous tumor volume (in mm^3^) over time (in days). Tumor growth kinetics showing significant reduction in tumor volume following glucocorticoid receptor (GR) blockade (*n* = 15, unpaired two-tailed *t* test on day 13, *p* = 0.0008, day 15, *p* = 0.0001 and day 17, *p* < 0.0001; two-way repeated-measures ANOVA with Geisser–Greenhouse correction to compare tumor-growth curves, *p* < 0.0001). **b** Flow cytometric analysis of tumor-infiltrating NK cells demonstrating increased production of effector cytokines TNFα, IFNγ, perforin, along with enhanced expression of activation receptors DNAM1, NKG2D, CD69, and decreased expression of inhibitory receptor KLRG1 in the GR-blocked group compared with vehicle-treated controls (Data are expressed as mean ± SEM, *n* = 12, unpaired two-tailed *t* test). **c** Heatmap showing the transcriptomic profile of tumor-infiltrating NK cells isolated from GR-blocked versus vehicle-treated mice. GR inhibition upregulated key effector-associated genes, while suppressing inhibitory or stress-responsive transcripts. **d** Gene set enrichment analysis (GSEA) of differentially expressed genes in tumor NK cells after GR blockade. Upregulated pathways included acute inflammatory response, type II immune response, interleukin production, and adaptive immune activation, whereas steroid catabolism and p38/MAPK signaling were suppressed. **e** GSEA enrichment plot highlighting the activation of the “acute inflammatory response” hallmark gene set in GR-blocked tumor NK cells (NES = 1.7, *p* = 0.005). **f** Pathway activity scores derived from PROGENy analysis showing significant restoration of PI3K signaling in NK cells following GR blockade (*p* = 0.035, Wilcoxon test). **g** Left panel: experimental schematic for intravenous LLC-OVA lung metastasis model. Tumor-bearing mice were treated with mifepristone or vehicle as described. Right panel: Representative quantification of lung tumor area showing significant reduction of lung tumor burden following GR (glucocorticoid receptor) inhibition (*n* = 6, unpaired two-tailed *t* test). **h** Flow-cytometric quantification of IFNγ-producing NK cells, degranulating NK cells (CD107a⁺), and DNAM1-expressing NK cells isolated from lung tumors of mice treated with vehicle (Lung tumor NK) or mifepristone (Lung tumor NK, GR blocked). (Data are expressed as mean ± SEM, *n* = 6; unpaired two-tailed t-test)

In glucocorticoid-rich lung TME, tumor-infiltrating NK cells respond to glucocorticoids through the nuclear receptor GR encoded by *NR3C1*. We hypothesize that inhibiting this receptor may reduce the glucocorticoid responsiveness of lung tumor NK cells and increase their cytotoxic activities. To test this, we subcutaneously injected the LLC-OVA cell line into syngeneic C57BL/6 J mice and allowed them to develop tumors. Experimental mice received the GR inhibitor mifepristone (60 mg/kg body weight, administered by oral gavage on alternate days) and control mice received vehicle (olive oil). GR blocked mice exhibit a significant reduction in tumor progression over time compared to vehicle-treated mice (Fig. 3a). After 18 days, immunophenotyping of tumor-infiltrating NK cells revealed upregulation of key activation markers, including TNFα, IFNγ, Perforin, DNAM1, NKG2D, and CD69, together with downregulation of the inhibitory receptor KLRG1, indicating enhanced recruitment of NK cells with greater cytotoxic potential in GR-blocked mice (Fig. 3b). GR inhibition also elevated the TNFα and IFNγ-expressing CD4⁺ and CD8⁺ T cells, consistent with heightened effector activity (Supplementary Fig. 4a). Moreover, arginase-expressing (anti-inflammatory M2-type) macrophages were reduced in GR-blocked tumors, suggesting a shift toward a more pro-inflammatory immune milieu (Supplementary Fig. 4b). In contrast, the cytotoxic profile of splenic NK cells and CD4⁺/CD8⁺ T cells remained unchanged between GR-blocked and vehicle-treated groups, indicating that GR blockade reinvigorates tumor-resident glucocorticoid-suppressed T and NK cells rather than activating systemic immunity. (Supplementary Fig. 4c, d).

Comparative transcriptomics analyses of tumor-infiltrating NK cells show a significant difference in GR-inhibited NKs vs the control group (Fig. 3c, Supplementary Fig. 4e, Supplementary Table 5). In GSEA biological pathway analysis we observed the upregulation of acute inflammatory response, type 2 immune response, higher expression of pro-inflammatory cytokines including IL1 and IL18 and activation of ERK cascade (relates to NK cell activation) in GR-inhibited tumor infiltrating NK cells, whereas GR inhibition downregulates the P38MAPK cascade (stress-activated pathway) and negatively influences steroid catabolic process (Fig. 3d, e), We also observed a marked upregulation of the PI3K signaling cascade in GR-inhibited tumor-infiltrating NK cells, indicating restoration of key activation pathways suppressed under glucocorticoid exposure (Fig. 3f). Comparative transcriptomic analysis of tumor-infiltrating NK cells from GR-inhibited versus control mice revealed distinct transcriptional reprogramming across activation, cytotoxicity, inhibition, and chemotaxis modules (Supplementary Fig. 4f). Consistent with this activation profile, GR inhibition also diminished hypoxia-associated transcriptional program in tumor-infiltrating NK cells. Expression of key hypoxic regulators was markedly reduced, indicating alleviation of glucocorticoid-driven hypoxic stress (Supplementary Fig. 4g). Collectively, this transcriptional shift highlights that GR inhibition restores NK-cell activation and effector programs while diminishing inhibitory signaling, thereby favoring a more cytotoxic phenotype within the lung TME.

In the lung metastasis model, inhibition of the GR significantly reduces the lung colonization of LLC-OVA cells (Fig. 3g, Supplementary Fig. 5a). GR inhibition led to enhanced activation of tumor-infiltrating NK cells, which showed increased expression of IFNγ, CD107a, and DNAM1 compared with controls (vehicle-treated mice) (Fig. 3h). A similar phenotype was observed in tumor-infiltrating CD4⁺ and CD8⁺ T cells, with GR-blocked mice showing elevated frequencies of IFNγ and TNFα-producing T cells relative to vehicle-treated counterparts (Supplementary Fig. 5b). Consistent with findings from the subcutaneous tumor model, splenic NK cells and T cells did not exhibit significant changes in cytotoxicity, indicating that GR inhibition selectively reinvigorates intratumoral immune effector function without altering systemic immunity (Supplementary Fig. 5c, d). These findings suggest that glucocorticoids negatively influence the cytotoxicity of tumor-infiltrating NK and T cells and exacerbate hypoxic stress. Blocking glucocorticoid signaling reinstates the cytotoxic function of NK and T cells and reduces hypoxic stress signaling.

### Cortisol-resistant CEACAM5-specific CAR-NK cells kill cancer cells in the presence of glucocorticoids

**Fig. 4:**
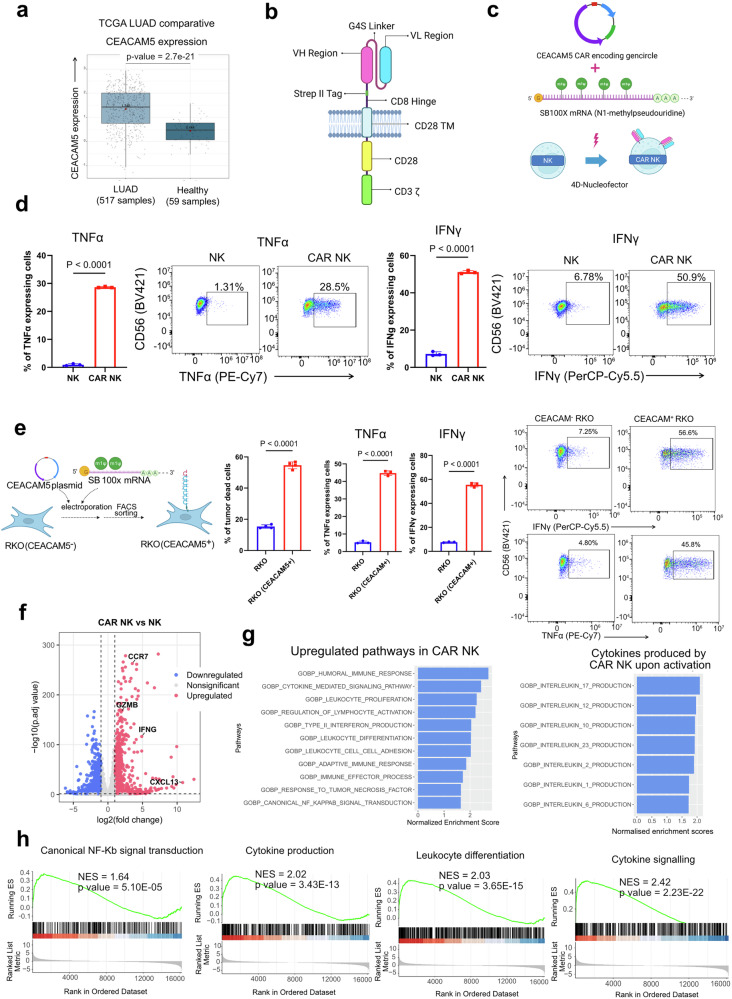
CEACAM5-specific CAR-NK targets CEACAM5-expressing cancer cell lines. **a** The upregulation of CEACAM5 expression in TCGA lung adenocarcinoma samples and normal tissue. The normalized expression of CEACAM5 in LUAD patient samples (*n* = 517) has been compared with healthy donors (*n* = 59 samples). **b** Schematic representation of CAR architecture. CAR featuring the intracellular signaling domains of CD28 and CD3ζ; the scFv is based on the variable-domain structure specific to the CEACAM5 antigen. The illustration was created using BioRender. **c** The generation of CEACAM5 CAR-NK through Sleeping Beauty technology. The GenCircle (a miniplasmid prepared by GenScript Biotech) encodes the CEACAM5-specific CAR sequence. The SB100X mRNA (which encodes Sleeping Beauty transposase) and the GenCircle have been co-transfected in the NK92 cell line in the 4D-Nucleofector. The illustration was created using BioRender. **d** CEACAM5-expressing A549 cells (target cells) have been cocultured either with CAR-NK cells or NK92 cells (effector cells) at a 1:1 ratio for 24 hours. The percentages of TNFα- and IFNγ-expressing NK-92 and CAR-NK cells were plotted based on FACS analysis after 24 hours of coculture. Gating: All cells>Singlets>Live cells> CD56 + TNFα+ or CD56 + IFNγ + . The flow cytometry plot showing the expression of effector cytokines IFNγ and TNFα by CAR-NK and NK cells in the presence of the CEACAM5 + A549 cell line. (Data are expressed as mean ± SEM, *n* = 3, unpaired two-tailed *t* test). **e** The making of the CEACAM5 + RKO cell line from the CEACAM5- RKO cell line. We co-transfected the CEACAM5-encoding plasmid and the Sleeping Beauty SB100x mRNA via electroporation. The CEACAM5 + RKO cells have been FACS sorted. CAR-NK cells have been cocultured with CEACAM5 + RKO and CEACAM5- RKO cell lines for 24 hours. The percentage of RKO cell death was quantified by flow cytometry. The percentage of TNFα- and IFNγ-expressing CAR-NK cells was plotted after 24 hours of coculture, based on flow cytometry analysis. Gating: All cells>Singlets>Live cells>CD56+IFNg+ or CD56+ TNFa + . The flow cytometry plot shows the percentage of IFNγ- and TNFα-CAR-NK cells after coculture with CEACAM5+ and CEACAM5- RKO cell lines. (Data are expressed as mean ± SEM, *n* = 3, unpaired two-tailed *t* test). The illustration was created using BioRender. **f** Differentially expressed genes derived from a transcriptomics study of activated CAR-NK cells. Volcano plot showing differentially expressed genes in CAR-NK compared with NK-92 after coculture with the CEACAM5 + A549 cell line. The significantly upregulated genes have been plotted in red, and the significantly downregulated genes have been plotted in blue. **g** Upregulated pathways in CAR-NK after culturing with CEACAM+ tumor cells upon GSEA analysis on differentially expressed genes in CAR-NK. Significantly upregulated immune pathways (*p* < 0.05) are shown after GSEA enrichment analysis. The bar plots show the major cytokines produced by activated CAR-NK upon co-culture with CEACAM5+ tumor cells. The gene ontology biological pathway analysis (GOBP) upon GSEA reveals the cytokines produced by CAR-NK cells. **h** Major upregulated pathways in activated CAR-NK obtained from GSEA analysis. Enrichment plots showing upregulated pathways from gene set enrichment analysis (GSEA) (*p* value < 0.05). The ranked list of genes (*x* axis) and the enrichment score (ES) (*y* axis) have been plotted. A positive ES indicates that the top-ranked genes in the activated CAR-NK cells

To translate the concept of glucocorticoid-mediated immune suppression in the lung TME, here we aim to generate lung cancer-specific glucocorticoid-resistant CAR-NK cells for immunotherapy. Reanalyzing the Cancer Genome Atlas (TCGA) dataset, we found CEACAM5 (Carcinoembryonic antigen-related cell adhesion molecule 5) shows significantly higher expression in lung tumors compared to normal lungs and drives the progression of non-small-cell lung cancer by promoting cell proliferation and migration (Fig. 4a).^25,26^ Therefore, we have engineered the NK-92 cell line to generate CEACAM5-specific cortisol-resistant CAR-NK cells. We used a CAR construct sequence specific to the CEACAM5 antigen, as reported in previous research. Using that sequence, we prepared a mini plasmid construct (GenCircle) with the help of GenScript (https://www.genscript.com/). Co-transfection of hyperactive Sleeping Beauty Transposase encoding SB100X mRNA and CEACAM-CAR encoding GenCircle into the NK-92 cell line creates CAR-NK cells (Fig. 4b, c).^27,28^ The expression of CAR is stable over time (Supplementary Fig. 6). The CEACAM5-specific CAR-NK cells, for 24 hours co-culture with A549 cell line, show significant cytotoxicity against CEACAM5 overexpressing A549 lung tumor cell line with elevated expression of IFNγ and TNFα (Fig. 4d). To check the specificity of CEACAM5-specific CAR-NK cells, we chose the CEACAM5 negative RKO cell line and created a CEACAM5 overexpressing RKO cell line (Fig. 4e). CEACAM5-specific CAR-NK cells were efficient in killing CEACAM5-overexpressing RKO cells but did not show any cytotoxic effect against CEACAM5-negative RKO cell lines (Fig. 4e). Transcriptomic analysis of CEACAM5-specific CAR-NK cells co-cultured with A549 cells demonstrates CAR-NK cell activation through the NF-κB signaling pathway, along with the enrichment of various pro-inflammatory cytokines, including IL-17, IL-12, IL-23, IL-1, and IL-6 production related gene programs. This CAR-specific activation promotes NK cell proliferation and enhances the expression of adhesion markers (Fig. 4f–h, Supplementary Table 6).

**Fig. 5:**
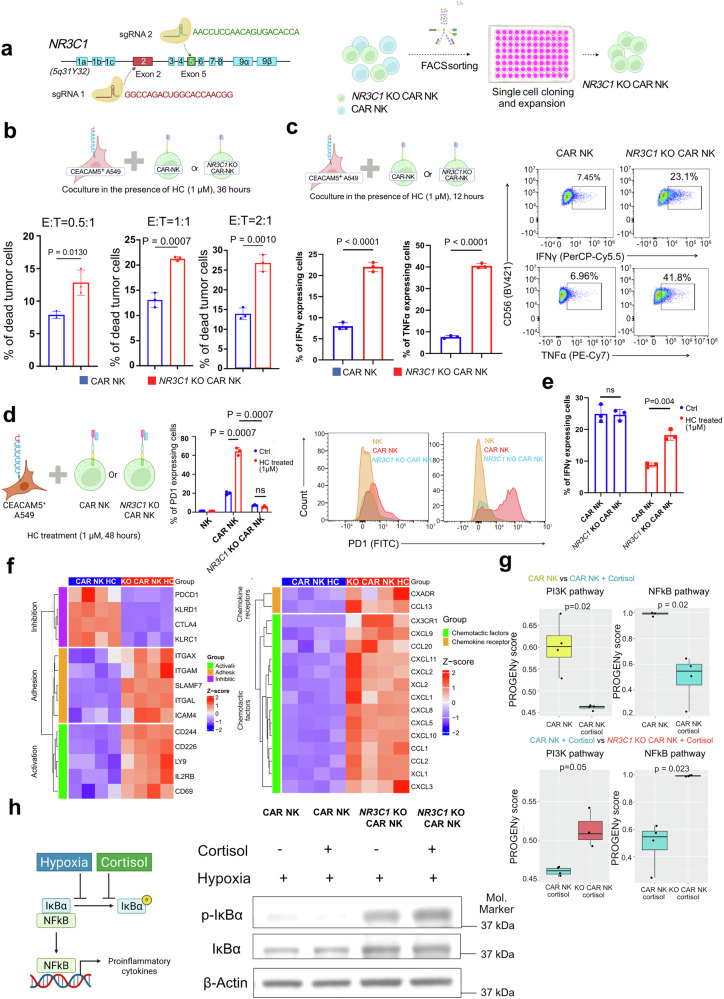
Glucocorticoid-resistant CEACAM5-specific CAR-NK cells eliminate tumor cells in the presence of glucocorticoid. **a** The diagram shows the structure of the glucocorticoid receptor (*NR3C1*) gene. The guide RNAs have been designed for exon 2 and exon 5 of the *NR3C1* gene. After the CRISPR knockout, the cells were sorted into a 96-well plate for single-cell cloning and expansion. After a month, a randomly selected clone was subjected to Sanger sequencing to identify *NR3C1*-KO cells in exons 2 and 5. *NR3C1*-KO cells are cortisol-resistant CAR-NKs. The illustration was created using BioRender. **b** Killing of CEACAM5 + A549 cells by *NR3C1*-KO CAR-NKs (cortisol resistant) and CAR-NKs after 36 hours of coculture in the presence of 1 micromolar of hydrocortisone (HC, cortisol) in different effector: target (E:T) ratios. All cells>Singlets>BFP+ cells (A549 cells) > dead cells. (Data are expressed as mean ± SEM, *n* = 3, unpaired two-tailed *t* test). The top illustration was created using BioRender. **c** *NR3C1*-KO (Cortisol-resistant) CAR-NKs and conventional CAR-NKs are cocultured with CEACAM5 + A549 cells for 12 hours in the presence of 1 micromolar hydrocortisone (HC, cortisol). The percentages of IFNγ- and TNFα-expressing CAR-NK cells and *NR3C1*-KO cells have been plotted based on flow cytometry analysis after 12 hours. The flow cytometry plot showing the expression of IFNγ and TNFα by CAR-NK and glucocorticoid-resistant (*NR3C1*-KO) CAR-NK after 12 hours of coculture with CEACAM5 + A549 cell line in the presence of one micromolar hydrocortisone (HC, cortisol) (Data are represented as mean ± SEM, *n* = 3, unpaired two-tailed *t* test). Gating: All cells>Singlets>Live cells>CD56 + IFNγ+ or CD56 + TNFα + . The top illustration was created using BioRender. **d** *NR3C1*-KO (Cortisol-resistant) CAR-NK and CAR-NK have been cocultured with CEACAM5 + A549 cell line in the presence of 1 micromolar hydrocortisone (HC, cortisol) for an extended time (48 hours), and the percentage of PD1 expressing cells was determined after flow cytometry analysis. (Data are represented as mean ± SEM, *n* = 3, unpaired two-tailed *t* test). The illustration was created using BioRender. **e** After 48 hours of coculture, the percentage of IFNγ-expressing CAR-NK cells and *NR3C1*-KO (Cortisol-resistant) CAR-NK cells was obtained from flow cytometry analysis. (Data are represented as mean ± SEM, *n* = 3, unpaired two-tailed *t* test). **f** Heatmaps illustrating transcriptomic profiles of key functional gene modules in CAR-NK and *NR3C1*-KO CAR-NK after co-culturing with CEACAM5 + A549 cells in the presence of hydrocortisone (HC, cortisol). *NR3C1*-KO CAR-NK cells retained high expression of activation and adhesion genes (*CD69*, *IL2RB*, *ITGAM*, *ITGAL*, *CD226*) and low expression of inhibitory markers (*PDCD1*, *CTLA4*). Chemokine receptors and chemokine factors were also upregulated in *NR3C1*-KO CAR-NK cells even under hydrocortisone treatment. **g** PROGENy pathway analysis showing downregulation of PI3K and NF-κB signaling pathways in activated CAR-NK cells upon cortisol exposure, both of which were in operation in *NR3C1*-KO CAR-NK cells under the same conditions (Wilcoxon test). Cells were activated using A549 cells expressing the CEACAM5 antigen. **h** Left: schematic illustrating hypoxia- and cortisol-mediated suppression of the NF-κB pathway in CAR-NK cells. Right: CAR-NK and *NR3C1*-KO (cortisol-resistant) CAR-NK cells were co-cultured under CoCl_2_-induced hypoxia ± cortisol. Cells were activated using A549 cells expressing the CEACAM5 antigen. NK cells were harvested and analyzed by immunoblotting for total IκBα and phosphorylated IκBα (p-IκBα). Combined hypoxia and cortisol markedly reduced IκBα phosphorylation in CAR-NK cells, indicating impaired NF-κB activation. In contrast, *NR3C1*-KO CAR-NK cells maintained IκBα phosphorylation under both hypoxic and cortisol-rich conditions, demonstrating functional resistance to cortisol-mediated suppression of NF-κB signaling. The nearest molecular ladder (molecular marker) position is shown. The illustration was created using BioRender

We further generated glucocorticoid-resistant CAR-NK cells by knocking out the *NR3C1* gene encoding the GR. CRISPR-mediated knock-out in exon 2 (encoding the N-terminal domain) and exon 5 (encoding ligand-binding domain) of the *NR3C1* gene disrupts the structure and ligand-binding ability of the distorted GR (Fig. 5a). To select complete *NR3C1* knock-out CAR-NK cells, we sorted them and expanded the single clones. Sanger sequencing confirms the knockout of *NR3C1* in selected clones (Fig. 5a). In the presence of cortisol (hydrocortisone, 1 µM), cortisol-resistant CAR-NK cells (*NR3C1* knockout) exhibit enhanced cytotoxic activity against the A549 cell line compared to conventional CAR-NK cells (Fig. 5b). This increased cytotoxicity is accompanied by sustained killing activity and upregulated expression of IFNγ and TNFα (Fig. 5c). After 48 hours of co-culture in the presence of cortisol (hydrocortisone, 1 µM), CAR-NK cells exhibited a significant increase in the exhaustion marker PD-1, impairing their killing activity. In contrast, the glucocorticoid-resistant CAR-NK cells showed no signs of exhaustion and maintained their cytotoxic activity (Fig. 5d, e). Transcriptomics study of glucocorticoid-resistant CAR-NK cells (*NR3C1*-KO CAR-NK) and CAR-NK cells obtained from cortisol-rich (1 µM) A549 co-culture, shows the downregulation of all glucocorticoid responder genes (e.g., *AREG, TSC22D3, FKBP5*) as we found from our initial experiment in glucocorticoid-resistant CAR-NK compared to CAR-NK (Supplementary Fig. 7a–c, Supplementary Table 7). GSEA pathway analysis reveals upregulation of cytotoxicity and activation of multiple pathways, including the NF-κB inflammatory response pathway, the IL-6/JAK-STAT3 pathway, and the IL-2/STAT5 signaling pathway (Supplementary Fig. 7d). However, these pathways are downregulated in the presence of cortisol in CAR-NK cells. These findings indicate that glucocorticoids inhibit CAR-NK cell function, whereas NR3C1-KO CAR-NK cells retain their killing activity despite the presence of glucocorticoids. Furthermore, transcriptomics study shows the upregulation of adhesion markers (*ITGAX, ITGAM, SLAMF7, ITGAL, ICAM4*), activation markers (*CD244, CD226, LY9, IL2RB, CD69*) and downregulation of inhibition markers (*PDCD1, KLRD1, CTLA4, KLRC1*) in cortisol-resistant CAR-NK cells with compared to CAR-NK cells (Fig. 5f). We also found elevated expression of chemokine receptor (*CX3CR1* and *CXADR*) and multiple chemotactic factors in glucocorticoid-resistant CAR-NK cells, which indicates its better potential to recruit other immune cells in lung TME compared to CAR-NK cells in the presence of glucocorticoid (Fig. 5f, Supplementary Table 7).

To delineate the mechanistic basis of cortisol-induced CAR-NK cell dysfunction, we performed PROGENy pathway analysis of transcriptomic data from activated CAR-NK and *NR3C1*-KO CAR-NK cells exposed to cortisol. Remarkably, two key regulators of NK-cell activation and cytotoxicity, PI3K-AKT and NF-κB signaling, were markedly suppressed in CAR-NK cells following cortisol exposure (Fig. 5g, top). In contrast, *NR3C1*-KO CAR-NK cells maintained (and effectively restored) PI3K-AKT and NF-κB pathway activity despite cortisol treatment (Fig. 5g, bottom). Consistently, transcriptional profiling revealed that PI3K pathway inhibitory genes were selectively upregulated in cortisol-treated CAR-NK cells, whereas their expression remained largely unaltered in *NR3C1*-KO CAR-NK cells (Supplementary Fig. 7e), indicating a GR-dependent suppression of PI3K activity. To further validate these findings at the protein level, we performed western blot analysis, which confirmed a reduction of phosphorylated AKT (p-AKT s473) and phosphorylated IκBα (p-IκBα Ser 32/36) in cortisol-treated activated CAR-NK cells, reflecting an impaired activation of both the PI3K-AKT and NF-κB pathways (Supplementary Fig. 7f). Notably, this inhibition was absent in cortisol-treated activated *NR3C1*-KO CAR-NK cells (Supplementary Fig. 7f, Supplementary Table 8), demonstrating that loss of GR signaling preserves these essential activation cascades under glucocorticoid stress. Together, these results reveal that cortisol directly hinders NK-cell effector functions by repressing PI3K-AKT and NF-κB signaling, and that *NR3C1* deletion effectively restores these pathways, conferring steroid resistance. Given our earlier observations that cortisol exacerbates hypoxic stress in NK cells, we next examined the transcriptomic landscape of activated CAR-NK and *NR3C1*-KO CAR-NK cells under hypoxia and combined hypoxia + cortisol conditions (Supplementary Fig. 7g, Supplementary Table 9). Both hypoxia alone and combined hypoxia with cortisol markedly attenuated NF-κB pathway activity in CAR-NK cells by reducing phosphorylated IκBα level (Fig. 5h, Supplementary Fig. 7h). Strikingly, *NR3C1*-KO CAR-NK cells preserved robust NF-κB signaling under these conditions (Fig. 5h; Supplementary Fig. 7i). This resilience suggests that genetic ablation of the GR shields CAR-NK cells from hypoxia-induced signaling collapse, enabling them to sustain NF-κB-mediated activation and cytotoxic competence even within the steroid-rich TME.

### In vivo efficacy of cortisol-resistant CAR-NK cells in a steroid-rich lung tumor model

**Fig. 6:**
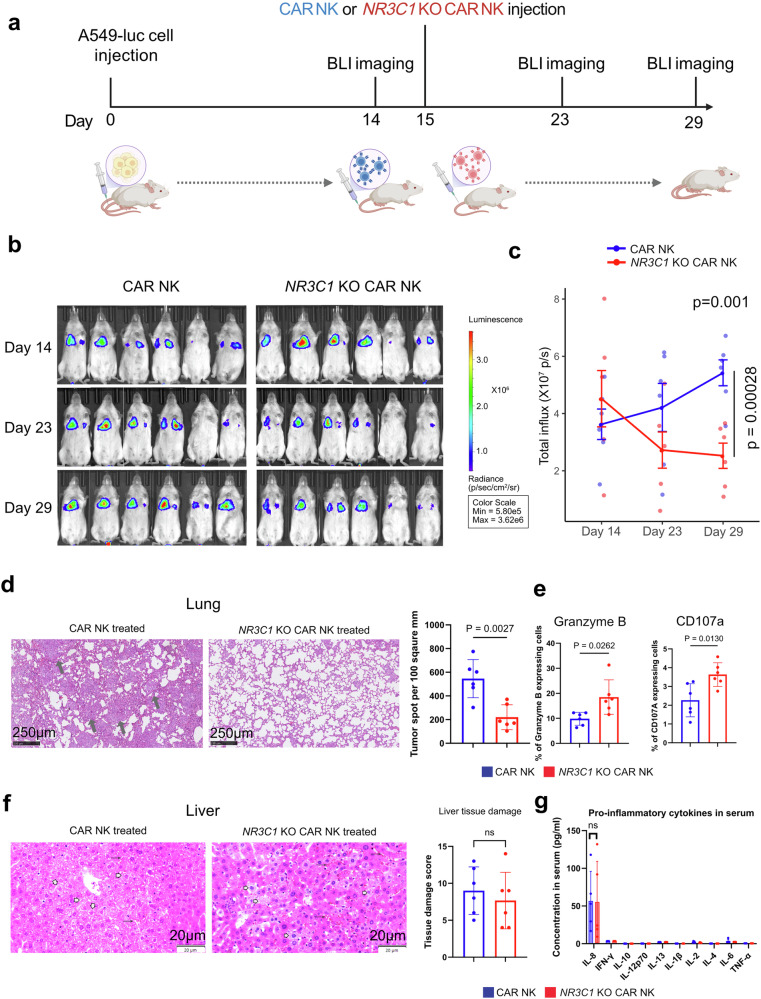
Cortisol-resistant CAR-NK cells exhibit enhanced anti-tumor efficacy in vivo. **a** Schematic representation of the experimental workflow. 12 NSG SGM3 mice were intravenously injected with 1 × 10⁶ A549-luciferase (A549-luc) lung cancer cells on day 0 to establish lung metastases. On day 15, tumor-bearing mice received 1 × 10⁷ CEACAM5-specific CAR-NK or *NR3C1*-KO CAR-NK cells via tail vein injection. Bioluminescence imaging (BLI) was performed on days 14, 23, and 29 to monitor tumor progression. The illustration was created using BioRender. **b** Representative serial BLI images of mice treated with CAR-NK (left) or *NR3C1*-KO CAR-NK (right) showing progressive tumor regression in the *NR3C1*-KO CAR-NK group across 14–29 days post-injection. **c** Quantification of total bioluminescence flux (photons s⁻¹) from lung regions over time shows reduced tumor burden in mice treated with *NR3C1*-KO CAR-NK cells compared with CAR-NK controls (*n* = 6). Tumor-growth curves differed significantly between groups (two-way repeated-measures ANOVA, Geisser–Greenhouse correction; Group×Time interaction *p* = 0.00028, unpaired two-tailed *t* test on day 29, *p* = 0.001). **d** Representative H&E-stained lung sections from CAR-NK and *NR3C1*-KO CAR-NK-treated mice. Tumor foci (arrows) were abundant in CAR-NK-treated lungs but were less abundant in the *NR3C1*-KO group. Quantification of tumor burden confirms a significant reduction in metastatic burden in the *NR3C1*-KO CAR-NK-treated group compared to the CAR-NK-treated group. (Data are represented as mean ± SEM, *n* = 6; unpaired two-tailed *t* test, *p* = 0.0027). Scale bar, 250 µm. **e** Flow cytometric analysis of tumor-infiltrating NK cells showing higher frequencies of CD107a⁺ and granzyme B⁺ cells in *NR3C1*-KO CAR-NK treated mice compared with CAR-NK treated controls (Data are represented as mean ± SEM, *n* = 6; unpaired two-tailed *t* test). (Gating strategy: All cells>Singlets>Live cells>hCD56+CAR + >hCD56 + CD107a+ or hCD56+GranzymeB+). **f** Representative H&E micrographs of liver tissue from CAR-NK (left) and *NR3C1*-KO CAR-NK-treated mice. Liver tissue sections showing vacuolated hepatocytes (white arrowheads) and congested sinusoids (arrows). Overall liver tissue damage has been scored based on vacuolated hepatocytes, sinusoidal and venous congestion, Kupffer-cell activation, and mononuclear infiltration. (Data are represented as mean ± SEM, *n* = 6; unpaired two-tailed *t* test, ns not significant). **g** Concentration (in pg/ml) of different proinflammatory cytokines in serum derived from CAR-NK-treated mice and *NR3C1*-KO CAR-NK-treated mice at day 30. (Data are represented as mean ± SEM, *n* = 5; unpaired two-tailed t test, ns not significant)

The superior in vitro cytotoxicity of cortisol-resistant (*NR3C1*-KO) CAR-NK cells prompted us to assess their anti-tumor efficacy in vivo. To establish an experimental lung metastasis model, we intravenously injected 1 × 10⁶ firefly luciferase-labeled A549 cells into NSG SGM3 mice (*n* = 6 in each group) (Fig. 6a). Bioluminescent imaging (BLI) confirmed lung colonization after 14 days. Mice were then randomly divided into two groups: one group received 1 × 10⁷ conventional CAR-NK cells, and the other 1 × 10⁷ *NR3C1*-KO CAR-NK cells on day 15 (Fig. 6a). To mimic the steroid-rich TME and to promote NK-cell persistence, both groups were co-treated with dexamethasone (10 mg kg^−1^) and recombinant human IL-2 (10,000 U per mouse) every other day. Longitudinal BLI monitoring revealed a significant reduction of tumor growth in the *NR3C1*-KO CAR-NK cell-treated group compared to the CAR-NK-treated group (Fig. 6b, c). Histopathological examination revealed significantly less tumor burden in the cortisol-resistant CAR-NK treated group (Fig. 6d). Flow-cytometric analyses of lung tissues showed that *NR3C1*-KO CAR-NK cells maintained significantly higher expression of Granzyme B and CD107a, consistent with sustained cytotoxic activation under glucocorticoid exposure (Fig. 6e). Despite the functional advantage, the abundance of CAR-NK and *NR3C1*-KO CAR-NK cells in the lung remained comparable between groups, as did their distribution in blood, bone marrow, liver, and spleen (Supplementary Fig. 8). Liver histology was comparable between CAR-NK- and *NR3C1*-KO CAR-NK-treated mice, with both groups showing preserved hepatic architecture and only mild changes, including vacuolated hepatocytes, sinusoidal and venous congestion, and minimal Kupffer-cell activation or mononuclear infiltration (Fig. 6f, Supplementary Table 10). Proinflammatory cytokine profiling (IFNγ, IL-1β, IL-2, IL-4, IL-6, IL-8, IL-10, IL-13, and TNFα) from CAR-NK and *NR3C1*-KO CAR-NK-treated mice revealed the presence of IL-2 and IL-8 in low concentrations (physiological range) in serum; however, we could not detect other proinflammatory cytokines (Fig. 6g).

## Discussion

Elucidating the mechanisms that drive dysfunction in tumor-infiltrating immune cells is essential for improving cancer immunotherapy. Although the immunomodulatory effects of steroids are well recognized, the contribution of local glucocorticoid signaling to NK-cell dysfunction in the lung TME remains underexplored. Here, we define a cortisol-rich immunosuppressive niche in lung TME and show that locally enriched cortisol drives NK-cell dysfunction and intensifies hypoxia-induced stress. We further delineate the cellular and metabolic routes that establish cortisol abundance, identify the signaling basis of cortisol-mediated suppression of NK-cell effector function, and translate these insights into therapy by engineering cortisol-resistant CAR-NK cells that retain anti-tumor activity in steroid-rich TME.

Tumor-infiltrating immune-cell dysfunction is multifactorial; Previous reports have implicated glucocorticoid signaling in shaping the immunosuppressive TME.^9,29,30^ Cancer cells have been reported to synthesize immunoregulatory glucocorticoids.^19^ However, no quantitative steroid profiling data were available. We explored the abundance and the impact of local glucocorticoids on lung tumor-infiltrating NK cells. While prior studies indicate that glucocorticoids hinder NK cell function by dysregulating activation, inhibitory markers, and modulating cytokine production,^31,32^ we found that glucocorticoids not only inhibit NK cell function directly but also affect the expression of hypoxia-regulated genes, likely exacerbating hypoxic stress in NK cells. Although the crosstalk between glucocorticoid and hypoxia-induced transcriptional responses has been reported in tissue homeostasis, the influence of local glucocorticoids on lung tumor-infiltrating NK cells has not been fully elucidated.^33,34^ While glucocorticoid abundance in the solid TME is documented, steroidogenesis and steroid recycling appear context-dependent.^8,19,35^ We demonstrate that de novo steroid biosynthesis and metabolic conversion regulate cortisol levels in the lung TME. Specifically, we delineated HSD11B1-driven cortisol recycling across stromal and immune populations, a mechanism previously implicated in cancer progression.^36,37^ De novo steroidogenesis implicated in T-cell mediated immune evasion appears distributed across immune and malignant compartments, accompanied by compartmentalized expression of downstream enzymes (CYP17A1, HSD3B2, CYP11B1) consistent with an active steroidogenic network.^4,38^ Notably, we identified CAFs and macrophages as critical hubs for steroid recycling, aligning with previously characterized HSD11B1-expressing iCAFs in pancreatic cancer.^39^

Single-cell transcriptomics of lung tumors suggests de novo steroidogenic capacity across malignant and immune compartments; however, spatially resolved steroid profiling coupled with immune mapping will be required to determine whether cortisol gradients govern NK-cell localization and function within the TME. Moreover, the upstream signals that induce immune-cell-mediated steroidogenesis in the TME and the determinants that drive CAFs and TAMs toward steroid-recycling states require deeper mechanistic dissection, including the extent to which inflammatory cues contribute to these phenotypes. CEACAM5 antigen is overexpressed by several tumor types, including lung cancer, colorectal cancer, breast cancer (especially ductal subtypes), and certain ovarian cancers.^26,40–42^ Overexpression of CEACAM5 on the tumor cell surface amplifies CAR-mediated signaling in CAR-NK cells and enables tumor-specific killing. However, as CEACAM5 is also expressed in healthy tissues, systematic profiling of antigen density and its relationship to CAR-NK activation kinetics will be crucial for predicting both therapeutic efficacy and potential off-target effects. In the lung metastasis model, *NR3C1*-KO CAR-NK cells achieved significantly improved tumor control despite systemic dexamethasone exposure, validating their therapeutic resilience in a steroid-rich microenvironment. CEACAM5-specific CAR-NK cells based on the NK-92 cell line can be easily revived and expanded from frozen stocks while maintaining CAR expression over time. Clinically, irradiated CAR-NK cells are employed to treat cancer. Several NK92-derived CAR-NK therapies currently in clinical trials show promising results with minimal side effects compared with CAR-T cells.^43–53^ These studies also demonstrate that CAR-NK cells may offer some advantages over CAR-T cells, including a reduced risk of cytokine release syndrome, neurotoxicity, and graft-versus-host disease. In our study, we did not detect any proinflammatory cytokines at elevated levels in serum from animals treated with CAR-NK or cortisol-resistant CAR-NK. This indicates a lower possibility of cytokine storm (cytokine release syndrome) in the host by CAR-NK itself. The use of the NK-92 cell line, though advantageous for reproducibility and clinical safety, limits long-term persistence and immune engagement.^54^ Future studies should extend this strategy to primary or induced pluripotent stem cell-derived NK cells, enabling evaluation of durability, memory potential, and trafficking in vivo.^55,56^

The translational implications of these findings suggest glucocorticoid resistance is a new design principle for cellular immunotherapies targeting solid tumors. CEACAM5 CAR-NK cells are suppressed by cortisol, whereas glucocorticoid-resistant CAR-NK cells sustain cytotoxicity in steroid-rich tumors, resulting in superior in vivo tumor control even during systemic dexamethasone treatment. This strategy is particularly relevant in clinical contexts where glucocorticoids are frequently administered to manage inflammation or immunotherapy-associated toxicities. Survival and biodistribution analyses demonstrated that *NR3C1*-KO CAR-NK cells persist in vivo for at least two weeks following intravenous administration, maintaining detectable levels in the bloodstream and distributing across multiple organs. Crucially, glucocorticoid-resistant CAR-NK cells preserved their ability to home to and infiltrate the TME, indicating that *NR3C1* deletion does not impair trafficking or tissue-surveillance programs. This widespread biodistribution was not associated with systemic inflammation or overt tissue pathology, and all treated mice remained clinically healthy, supporting a safety profile. Functionally, although both conventional and engineered CAR-NK cells reached the lung tumor, *NR3C1*-KO CAR-NK cells uniquely maintained potent cytotoxic activity within cortisol-rich, immunosuppressive niches. A single therapeutic dose of glucocorticoid-resistant CAR-NK cells induced striking clearance of lung tumors, highlighting their robustness under physiological stress and establishing a rationale for repeat dosing and combinatorial therapeutic strategies. Finally, the inclusion of both male and female mice was intentional to capture potential sex-associated biological variation, recognizing that such inclusion may introduce additional variability while improving the translational relevance of the findings. From a translational perspective, this approach may complement immune checkpoint blockade therapeutic approaches. Incorporating *NR3C1* deletion into CAR-based therapies could therefore enhance their performance in glucocorticoid-mediated immunosuppressive tumors, including lung, colon, breast, and pancreatic cancers.^4,6,57^

In conclusion, this study identifies local glucocorticoid signaling as a central and previously underappreciated mechanism of immune suppression in the lung TME. Quantitative steroid profiling and integrative single-cell analyses support the presence of a cortisol-rich niche in lung tumors, and functional experiments demonstrate that cortisol suppresses NK-cell cytotoxicity and amplifies hypoxia-associated stress through GR-dependent repression of NF-κB signaling. Deletion of the cortisol receptor protects therapeutic NK cells from steroid-mediated suppression, enabling sustained anti-tumor activity and improved tumor control in vivo in a steroid-rich microenvironment. Collectively, our work highlights intrinsic cortisol abundance in lung cancer and shows that engineering cell-intrinsic glucocorticoid resistance can help unlock the full therapeutic potential of NK-cell-based immunotherapies in cortisol-enriched solid tumors.

## Materials and methods

### Cell lines, primary cells, and culture conditions

Peripheral blood samples were collected from healthy donors with their informed consent. Peripheral blood mononuclear cells (PBMCs) were isolated through density gradient centrifugation using Ficoll-Paque Plus (GE Healthcare). We followed the protocol of NK cell enrichment from PBMC, as reported in previous studies.^58^ Freshly isolated PBMCs (3 × 10^6^ cells) were co-cultured with K562 cells irradiated with 100 Gy X-rays (0.5 × 10^6^ cells) in a 24-well tissue culture plate. The cultures were maintained in complete RPMI-1640 medium (RPMI media supplemented with 10% heat-inactivated serum, 1% PenStrep, 1 mM NaPyr (stock:100 mM), 10 mM HEPES (stock 1 M), and 50 μM B-ME (stock 50 mM)), supplemented with 10 U/ml recombinant human IL-2 (Peprotech, cat no 200-02). The medium was refreshed every 2–3 days with fresh medium containing 10 U/ml IL-2. After 7 days, the IL-2 concentration was increased to 100 U/ml, and 10 U/ml IL-15 was added. The medium was subsequently replaced every 2–3 days. This culture enriches NK cells from PBMCs with purity >90%. NK-92 cells (a human NK cell line) were a kind gift from Dr. Francesco Colucci, University of Cambridge. These cells have been maintained in RPMI medium supplemented with 10% heat-inactivated serum, 1% PenStrep, 1 mM NaPyr (stock: 100 mM), 10 mM HEPES (stock: 1 M), 50 μM B-ME (stock: 50 mM), and 100 U/ml IL-2. The media was replenished every 3 days. The K562 cell line was a kind gift from Dr. Andrew M Sharkey, University of Cambridge. The K562 cells are irradiated with 100 Gy of X-rays before being used as feeder cells. A549 (human lung carcinoma cell line) was a kind gift from April D’Arcy (Dr Brian Ferguson’s lab), University of Cambridge. The LLC-OVA cell line was a kind gift from Professor Klaus Okkenhaug at the University of Cambridge. RKO (human colorectal cell line) was a kind gift from the Stem Cell Institute, University of Cambridge. The adherent cells have been cultured in DMEM (Invitrogen) supplemented with 10% FBS, 1% penicillin-streptomycin, 1 mM NaPyr, 10 mM HEPES, and 50 μM B-ME.

### Bulk RNA-seq processing and analysis

RNA-Seq was performed on high-quality total RNA samples from different samples. RNA was extracted from human primary NK (*n* = 4), human primary NK treated with cortisol (*n* = 4), activated human primary NK (co-cultured with K562 for 6 hours) (*n* = 4), and activated human primary NK treated with cortisol (*n* = 3). For CAR-NK and glucocorticoid-resistant CAR-NK-related RNA sequencing, RNA was extracted from NK-92 (*n* = 4), CAR-NK (*n* = 4), CAR-NK treated with 1 micromolar cortisol (*n* = 4), *NR3C1*-KO CAR-NK (*n* = 4), and *NR3C1*-KO CAR-NK treated with 1 micromolar cortisol (*n* = 4) (all cells have been collected from co-cultured with the A549 cell line). For glucocorticoid treatment, cells were treated with one micromolar cortisol (hydrocortisone, h0888-1g, Sigma-Aldrich). In mouse experiments, NK cells have been isolated from subcutaneous tumors. cDNA libraries were constructed, and Sequencing was performed in paired-end mode on an Illumina Novaseq 6000 platform, yielding an average of 30 million reads per library. Raw sequencing reads (FASTQ files) are pre-processed, followed by alignment to the reference genome gencode M32 (Mouse genome GRCm39) or human genome GRCh38 using the alignment tool hisat2 (version 2.2.1).^59^ The sam files are further inspected, filtered, and processed using samtools (version 1.13).^60^ HTseq counts obtained from bam files are annotated and subjected to DESeq2 (version 1.34.0) analysis in R (version 4.1.1).^61^ In the DESeq2 analysis, for each gene, a log2 fold change was computed, and significance was assessed using the Wald test statistic, yielding a *p* value and an adjusted *p* value. Differentially expressed genes across conditions were identified using a *p* adjusted value cutoff of 0.05. The volcano plot was constructed in R using differentially expressed genes identified in bulk RNA sequencing.

### Functional enrichment analysis

After differential expression analysis with DESeq2, genes were ranked by fold change, and then, using the fGSEA algorithm, GSEA was performed on all candidate gene sets in the Hallmark database to identify significantly altered pathways. The mouse collection M5: ontology gene sets, the human geneset H: hallmark gene sets, and C5: ontology gene sets from the Molecular Signatures Database (MSigDB) have been used to identify positively and negatively enriched pathways.^62^ All steps have been performed in R version 4.1.1.

### Confocal imaging of HSD11B1 in frozen lung tissue: staining and imaging

Optimal cutting temperature (OCT)-embedded frozen human lung cancer samples were sectioned and mounted on glass slides. Slides were fixed with 1% formalin solution (Sigma-Aldrich) in PBS for 10 minutes at room temperature. After fixation, slides were washed three times with wash buffer (PBS supplemented with 0.5% Tween-20) and permeabilized with 0.2% Triton X-100 in PBS for 10 minutes. This was followed by three additional washes with wash buffer. To block non-specific binding, sections were incubated with 10% goat serum (Invitrogen 31873) in wash buffer for 1 hour at room temperature. Slides were then incubated overnight at 4 °C in a humidified chamber with a rabbit anti-HSD11B1 Polyclonal antibody (Proteintech, 10928-1-AP; 1:100 dilution) prepared in wash buffer containing 5% goat serum. The following day, slides were washed three times with wash buffer (10 minutes per wash at room temperature), then incubated for 1 hour at room temperature in the dark with Alexa Fluor 488-conjugated goat anti-rabbit IgG secondary antibody (Abcam; 1:5000 dilution in 5% goat serum in wash buffer). After secondary antibody incubation, sections were washed three times with wash buffer and counterstained with DAPI (1:2000 dilution) for 10 minutes. Membranes were then stained with CellMask Deep Red according to the manufacturer’s protocol. Final washes were performed three times in wash buffer before slides were mounted using CitiFluor AF1 mounting medium. Imaging was performed using a confocal microscope (STELLARIS8, Leica, Wetzlar, Germany). Images were acquired using a frame size of 512 × 512 pixels in the violet (DAPI; 405 nm excitation, 420–470 nm emission), green (Protein of interest; 488 nm excitation, 495–545 nm emission), and the far-red (CellMask Deep red; 637 nm excitation, 645–700 nm emission) channel. Within the FOV, the upper and lower Z-positions were selected based on the green-channel emission signal, and images were acquired as Z-stacks with 0.5 µm inter-stack spacing.

### Generation of CAR-NK cells using Sleeping Beauty (SB) transposon system

To engineer CEACAM5-specific CAR, Sleeping Beauty (SB) transgene cassettes were used. It enables overexpression of second-generation CARs in the NK92 cell line, targeting human CEACAM5. All parental plasmids were custom-designed and obtained from VectorBuilder. For anti-CEACAM5 CAR expression in NK92 cells, the hMN-14 SB vector was synthesized into ultrapure GenCircles (Genscript Biotech). The SB100X mRNA with N1-methyl pseudouridine (m1Ψ) (Genscript Biotech) encoding the Sleeping Beauty transposon has been used to incorporate and stably express the CAR construct. All transgene cassettes, except SB100X, were regulated by the EF1α promoter, whereas SB100X was controlled by the CAG promoter. All electroporations were performed using a Lonza 4D-Nucleofector. For electroporation in the NK92 cell line and RKO cell line, 2 million cells were resuspended in nucleofection buffer and pulsed according to their respective code (For the NK-92 cell line SE kit was used, the nucleofection code was CA 137, and for the RKO cell line SE kit was used with the nucleofection code EN113). For making the CAR-NK 16.4 µL SE solution + 3.6 µL supplement, add 4.8ug of SB100X and 1ug of CEACAM5-specific CAR encoding plasmid. Following nucleofection, cells were immediately rescued with 70 μL of pre-warmed complete media (antibiotic-free) and incubated at 37°C for 20 minutes. Subsequently, cells were further diluted and cultured in complete media. CAR^+^ NK cells were stained with anti-strep-tag II antibody (FITC) and sorted by FACS.

### Construction of CAR

The anti-CEACAM5 single-chain variable fragment (scFv) comprises variable heavy (VH) and light (VL) domains derived from the hMN-14 clone. This CAR construct incorporates a Strep-tag II sequence (WSHPQFEK) and a flexible G4S2 linker, positioned between the CD8α hinge and the scFv domains. The introduction of the Strep-tag II sequence enables easy identification of CAR-positive cells in flow cytometry. The CD28 transmembrane domain is used in conjunction with the intracellular signaling domains of both CD28 and CD3ζ for effective signaling. The CEACAM5-specific CAR construct has been derived from the United States Patent Application Publication. No.: US 2003/0148409 A1 (Pub. Date: Aug. 7, 2003).

### CRISPR-mediated knockout of *NR3C1* in CAR-NK cells

The sgRNAs have been designed to delete exons 2 and 5 of the *NR3C1* gene. The sequence of guide RNA for exon 2 is 5’ GGCCAGACUGGCACCAACGG 3’ and the guide RNA for exon 5 is 5’ AACCUCCAACAGUGACACCA 3’. The nucleofection was carried out using the SE kit (nucleofector program CA137). Post nucleofection, the cells were sorted into a 96-well plate and clonally expanded. Only clones with complete knockout efficiency in both exon 2 and exon 5 of the *NR3C1* gene were used for downstream experiments. We have confirmed the knock-out efficiency using Sanger sequencing.

### Single-cell RNA sequencing, clustering, and visualization

The previously published, publicly available scRNA-seq datasets used in this study were as follows: GSE127465, GSE131907, and single-cell data from 103 patient samples and other datasets (GSE148071, KU_loom, GSE153935, GSE136246, GSE119911, GSE127465). The data were downloaded from NCBI GEO (https://www.ncbi.nlm.nih.gov/geo/) and processed using the standard scRNA-seq integration pipeline in Seurat.^63^ We also reanalyzed 318 lung cancer patient sample data from the Single-cell **Lu**ng **C**ancer **A**tlas dataset (https://luca.icbi.at/),^24^ we took the fibroblast population from this integrated scRNA seq dataset for in-depth analysis. To cluster the cells, we first identified the top 2000 most variable genes using the FindVariableFeatures function in Seurat. For cell visualization, we applied the Uniform Manifold Approximation and Projection (UMAP) algorithm to project the cells’ PCA space representation into two dimensions. Cell clustering in PCA space was performed with the shared nearest neighbor (SNN) algorithm, using Seurat V3’s FindNeighbors and FindClusters functions. We then visualized the resulting clusters in UMAP space with the DimPlot function. The differentially expressed genes in each cluster were identified with the FindAllMarkers function in Seurat. The clusters have been identified either by previous annotation or by the cluster identity predictor (CIPR) program and validated using marker gene expression from PangloDB.^64,65^ Gene expression levels were visualized using heatmaps, violin plots, and bar plots, created with the pheatmap package (version 1.0.12) (available at https://cran.r-project.org/web/packages/pheatmap) and the dittoSeq R package (version 1.2.4). Data scaling during visualization was performed automatically using the default settings of these packages.^66^

Cortisol response score, hypoxia response score, NK dysfunctional score, and NK effector score have been calculated using Seurat’s AddModuleScore function, using the cortisol-responsive gene list as the input feature set.

### Marker genes used for scRNA-seq cell-type and functional annotation

#### NK-cell exhaustion markers

*NKG2A (KLRC1), KIR2DL3, TIGIT, HAVCR2 (TIM3), PDCD1 (PD-1), LAG3, KLRG1, TIM3*

#### CD8+ T-cell exhaustion markers

*PDCD1, CTLA4, LAG3, HAVCR2, TIGIT, BTLA, CD244, CD160, KLRG1, C10orf54 (VISTA), TOX, TOX2, NR4A1, NR4A2, BATF, NFATC1, EOMES, ENTPD1 (CD39), NT5E (CD73), CXCL13, IL10*

#### Hypoxia-response signature genes

*HIF1A, DDIT4, PFKFB3, ALDOA, CXCR4, ETS1, ATF3, JUN, HSPA5*

#### NK-cell dysfunction markers

*NR4A1, NR4A2, NR4A3, SOCS1, NFATC1, CBLB*

#### NK-cell effector genes

*PRF1, GZMB, IFNG, TNF, NKG7, KLRK1, DNAM1, EOMES, STAT5A, STAT5B*

#### TAM subtype markers

*Complement-associated TAMs:*

*C1QA, C1QB, C1QC, C3AR1, C5AR1*

*Interferon-stimulated TAMs (ISG-TAMs*):

*ISG15, IFI6, IFIT3, CXCL9, CXCL10, STAT1, IRF7*

Lipid-associated macrophages (LAMs):

*APOE, APOC1, LPL, TREM2, CTSD, TYROBP*

M2-like TAMs:

*MRC1 (CD206), CD163, MSR1, IL10, SEPP1, F13A1*

#### CAF subtype markers

iCAF: *C3, CXCL12, IL6, PDPN, PTGS2*

Epi-CAF: *HLA-DRA, CD74*

dCAF: *PCNA, MCM2, TOP2A, MKI67*

myCAF: *MMP11, CDH11, POSTN, ACTA2, TAGLN, COL1A1*

#### Cytotoxicity of CAR-NK cells and glucocorticoid-resistant CAR-NK Cells in vitro

To determine the cytotoxicity of CAR-NK and glucocorticoid-resistant CAR-NK cells, a co-culture experiment was performed using a CEACAM5 + BFP+ cell line at different E:T ratios. The confluence of adherent cells was ~80%, and 100 K cells had been seeded for co-culture. After co-culture, the number of dead tumor cells was quantified using live-dead NIR or Propidium iodide staining and flow cytometry. The expression of IFNγ, TNFα, and granzyme B and CD107a expression by CAR-NK and glucocorticoid-resistant CAR-NK cells was determined using flow cytometry.

### Mice

In this study, all C57BL/6 J mice were handled and cared for according to the strict guidelines established by the UK Animals in Science Regulation Unit’s Code of Practice for the Housing and Care of Animals Bred, Supplied, or Used for Scientific Purposes and the Animals (Scientific Procedures) Act 1986 Amendment Regulations 2012. Experimental procedures were carried out under the authority of a UK Home Office Project License (PPLs: PP4938782 and P0AB4361E) and were approved by the institute’s Animal Welfare and Ethical Review Body to ensure compliance with ethical and welfare standards. All mice were housed in the Gurdon animal facility under specific pathogen-free conditions, with a 12-hour light and 12-hour dark cycle. The LLC-OVA cell line has been used to develop a subcutaneous tumor in C57BL/6 J mice. 1 million LLC-OVA cells were injected subcutaneously in each mouse; from there, half of the mice were treated with mifepristone (60 mg/kg body weight) every other day, and the other half of the mice were treated with vehicle (olive oil) through oral gavage for 18 days. The mifepristone concentration has been determined based on the previous literature.^2^ On the 19th day, mice were euthanized, and the tumor was processed to isolate NK cells. We studied the transcriptomics of tumor-infiltrating NK cells.

### Western blot antibodies

IκBα - Cell Signaling 4814 P, Phospho-IκBα (Ser 32/36) - Cell Signaling 9246, Akt (pan) (C67E7) Cell Signaling Rabbit mAb 4691, Phospho-Akt (Ser473) Cell Signaling 9271 were used.

### Tumor tissue processing

The tumors were mechanically dissociated and were then subjected to enzymatic digestion using a mixture of 1 mg/ml collagenase D (Roche), 1 mg/ml collagenase A (Roche), and 0.4 mg/ml DNase I (Sigma) in IMDM media containing 10% FBS, all incubated at 37 °C for 30–40 minutes. To halt collagenase activity, EDTA (5 mM) was introduced to all samples. The digested tissues were finally strained through 70 μm cell strainers (Falcon) to ensure uniformity in sample preparation. After tissue processing, samples with sufficient viable cells were analyzed by flow cytometry.

### In Vivo Testing of CAR-NK and *NR3C1*-KO CAR-NK cells in NSG mice

NSG-SGM3 mice (NOD.Cg-Prkdcscid Il2rgtm1Wjl Tg(CMV-IL3, CSF2, KITLG)1Eav/MloySzJ) were purchased from The Jackson Laboratory and used to establish breeding colonies, which were housed in individually ventilated cages and maintained in a pathogen-free facility at the Central Biomedical Services, University of Cambridge. All experimental procedures were approved by the United Kingdom Home Office under the Animal (Scientific Procedures) Act 1986 (PPL numbers: PP5753595 and PP1611558). To establish a lung metastasis model, mice were intravenously injected with 1 × 10⁶ firefly luciferase-labeled A549 lung adenocarcinoma cells in 100 μL PBS via the tail vein. After 14 days, tumor colonization was confirmed by bioluminescent imaging (BLI) using the IVIS Spectrum imaging system (PerkinElmer) following intraperitoneal injection of D-luciferin (150 mg/kg). Mice were then randomized into two treatment groups for a blinded experimental setup. The CAR-NK group received 1 × 10⁷ conventional CEACAM5-specific CAR-NK cells, *NR3C1*-KO CAR-NK group received 1 × 10⁷ *NR3C1*-deficient CAR-NK cells via intravenous injection on day 15. To mimic the steroid-rich TME and support NK-cell persistence, both groups received dexamethasone (10 mg/kg, intraperitoneally) and recombinant human IL-2 (10,000 U/ mouse) on alternate days for 14 days. Tumor progression was monitored longitudinally using BLI at defined time points (days 14, 23, and 29). At the endpoint, mice were euthanized, and lung, liver, spleen, bone marrow, and peripheral blood were harvested for flow cytometry, histopathology, and immunophenotyping. Flow cytometric analysis was performed using a Cytek Aurora. Lung and liver histology were evaluated on formalin-fixed, paraffin-embedded sections stained with hematoxylin and eosin (H&E).

### Cell sorting and RNA extraction

After processing the tumor samples into single-cell suspensions, they were incubated for 1 hour at 4 °C with anti-mouse CD45 (FITC-conjugated), anti-mouse CD3 (APC-conjugated), and anti-mouse NK1.1 (PE-Cy7-conjugated) antibodies, diluted in PBS containing 1% bovine serum albumin (BSA) and 2 mM EDTA. DAPI was added before sorting. The stained samples were then sorted using a BD FACSAria™ II cell sorter. Cells have been collected in serum, and RNA has been extracted using the RNeasy Mini Kit or the RNeasy Micro Kit (QIAGEN) based on the manufacturer’s protocol. In the case of NK cells sorted from a mouse tumor, the RNA was amplified using the SMARTer amplification technique before proceeding to RNA-seq.

### Collection of human lung tumor tissue

Human samples used in this research project were obtained from the Imperial College Healthcare Tissue Bank (ICHTB). ICHTB is supported by the National Institute for Health Research (NIHR) Biomedical Research Center based at Imperial College Healthcare NHS Trust and Imperial College London. ICHTB is approved by Wales REC3 to release human material for research (22/WA/0214), and the samples for this project, R24029, were issued from sub-collection reference numbers of each sample given in Supplementary table 1. PBMCs from healthy donors were obtained under a protocol approved by the Human Biology Research Ethics Committee of the University of Cambridge (HBREC2019.15).

### PROGENy pathway analysis

Pathway activity scores were inferred using the PROGENy R package (version 1.30). Briefly, differential expression analysis was performed on normalized RNA-seq data, and the resulting gene-level expression matrix was used as input. PROGENy was run with the “top 100” footprint genes per pathway and human model settings to compute weighted pathway activity scores for each sample or condition. All analyses regarding the PI3K AKT pathway and the NF-κB pathway have been performed from the RNA of activated CAR-NKs or cortisol-resistant CAR-NK cells. For activation, CAR-NK or cortisol-resistant CAR NK cells have been co-cultured with CEACAM5-expressing A549 cells. Unless otherwise specified, default parameters were applied. Resulting pathway scores were compared between groups using the Wilcoxon rank-sum test (two-sided), and adjusted P values were reported where applicable.

### Mass spectrometric steroid profiling

Steroid levels (cortisol, cortisone, corticosterone, and pregnenolone) were quantified in 34 lung tumor tissues. Comprehensive steroid profiling, including glucocorticoids, mineralocorticoids, androgens, estrogens, and progestogens, was subsequently performed in a subset of 20 lung tumor tissues. Stage-stratified cortisol analysis was performed in 22 tumors. Steroids were measured by liquid chromatography-tandem mass spectrometry (LC-MS/MS) using ultra-performance liquid chromatography (UPLC) coupled to triple-quadrupole mass spectrometry. Samples were enriched with isotopically labeled internal standards and were extracted using supported liquid extraction alongside a calibration curve of multiple steroids. Tissue samples were thawed, homogenized, enriched with isotopically labeled steroids as internal standards, and acetonitrile-extracted prior to LC-MS/MS analysis.^67^ Sample extracts were separated on a Waters Acquity I-Class system fitted with a Kinetex C18 column (150 × 2.1 mm; 2.6 μm). This was followed by Mass spectrometry analysis on a QTRAP® 6500+ System (AB Sciex, UK) (RRID: SCR_021833) using polarity switching electrospray ionization and multiple reaction monitoring, alongside a multi-steroid calibration curve (0.0025–10 ng) for precise steroid quantification, analyzed using MultiQuant software (AB Sciex, UK).^68^

### Flow cytometry

Flow cytometry was performed on a Cytek Aurora [Spectral Flow Cytometry] Cytometer, with all analysis done on FlowJo software (v10.9.0). Cells were stained for viability with LIVE/DEAD Fixable Near-IR or Live dead Ghost dye and blocked with Human TruStain FcX (Biolegend) or mouse TruStain FcX (Biolegend) before antibody staining. All gating strategies used in the analysis are shown in Supplementary Figs. 10 and 11. For antibody staining, cells were resuspended in FACS buffer (PBS with 2% FBS). Cells were fixed in IC Fixation Buffer (eBioscience) and washed in FACS buffer twice before acquisition. The following antibodies have been used in flow cytometry study in mice tumor tissue CD45 (Buv563), Ghost Dye UV 450, IFNγ (BUV737), CD161(NK1.1)(BUV805), CD3 (Spark Blue 550), TNF (Spark NIR 685), CD49b (APC-Fire 750), PD1 (PE), CD184 (CXCR4) (Brilliant Violet 711™), CD226 (DNAM1) (PE/Dazzle™ 594), KLRG1 (MAFA) (PE cy7), Granzyme B (FITC), Perforin (APC/Fire™ 750), CD152 (CTLA4) (Brilliant Violet 605™), NKG2D (APC). In humans, the antibodies were IFNγ (PerCP-Cy5.5), TNFα (PE-Cy7), CD56 (BV421), PD-1 (FITC), Granzyme B (APC), and CD107a (PE) (BioLegend).

### Measurement of liver tissue damage

Semi-quantitative tissue scoring was carried out depending on the visual field inspection of liver tissue sections stained by H and E from each group. Organ morphometrical analysis was carried out by giving a score based on the level of damage seen in each section in each group, according to the severity in the examined tissue: 0 = no lesions; 1 = mild (1 to 25%); 2 = moderate (26 to45%); 3= severe (>45%) as described previously,^69,70^ and then the scores were added to create a final total score (TLS) ranging from 0 to 18. Liver tissue sections were scored according to alterations in vascular changes, inflammation, Kupffer cell reaction, hepatocellular degeneration, necrosis, and anisokaryosis.^71^ Morphometry was carried out at the Image Analysis Unit, Department of Pathology and Clinical Pathology, Faculty of Veterinary Medicine, Sohag University.

### Statistical analysis

Statistical analyses were performed using unpaired two-tailed Student’s *t* tests, Wilcoxon rank-sum tests, or two-way ANOVA, as appropriate and as specified in the figure legends. Differentially expressed genes in bulk and single-cell RNA-seq datasets were identified using the statistical frameworks and default *P* value calculations implemented in the respective R packages. Sample sizes were determined based on power calculations, pilot experiments, or prior experience with variability in similar assays. Samples that failed quality control or experienced technical processing issues were excluded before analysis, and no other data were removed. Statistical significance was defined as *P* < 0.05 unless stated otherwise. Analyses were performed in GraphPad Prism 10 (GraphPad Software) or R, and schematic illustrations were generated using BioRender.com.

## Supplementary information

Supplementary Materials

Data Set 1

Data Set 2

Data Set 3

Data Set 4

Data Set 5

Data Set 6

Data Set 7

Data Set 8

Data Set 9

Data Set 10

## Data Availability

Newly generated bulk RNA seq data are deposited to EBI ArrayExpress with the accession numbers E-MTAB-16367, E-MTAB-16365, E-MTAB-16373, and E-MTAB-16381 (https://www.ebi.ac.uk/biostudies/arrayexpress). Previously published single-cell RNA-sequencing datasets analyzed in this study were obtained from NCBI GEO under the following accession numbers: GSE127465, GSE131907, GSE148071, GSE153935, GSE136246, GSE119911, and KU_loom, encompassing data from 103 NSCLC patient samples across multiple studies. All datasets were downloaded from the GEO repository (NCBI GEO; https://www.ncbi.nlm.nih.gov/geo/). In addition, we re-analyzed 318 lung cancer patient samples from the Single-cell Lung Cancer Atlas (LUCA) dataset (https://luca.icbi.at/). The other data supporting the study’s findings, along with the codes used for analysis, are available upon reasonable request.
