# Supplementary material for: Cortisol-resistant CAR-NK cells overcome steroid-induced immunosuppression in lung cancer
Source: Signal Transduct Target Ther. 2026 Apr 9;11:128. doi: 10.1038/s41392-026-02638-z (PMC13066642; doi:10.1038/s41392-026-02638-z)
Supplement: Supplementary file 1 — Supplementary Materials [file 41392_2026_2638_MOESM1_ESM.docx]

Supplementary Materials for

Cortisol-resistant chimeric antigen receptor (CAR)-NK cells overcome steroid-induced immunosuppression in lung cancer

**Authors:** Soura Chakraborty^1^, Jhuma Pramanik^1^, Gustavo Alviter-Raymundo^2^, Christopher J. Ward^1^, Sanu K. Shaji^1^, Yumi Yamashita-Kanemaru^1^, Fatma Abo Zakaib Ali^3,4^, Debasis Banik^5^, Ziwei Zhang^5^, Clara Veiga-Villauriz^1^, Natalie Z. M. Homer^6^, Joanna Simpson^6^, Sofia Laforest^6^, Shanlin Tong^1^, Qiuchen Zhao^1^, James Roy^7^, Muhammad Iqbal^1^, Andrew Conway Morris^1,8^, Michael A Chapman^7^, Rahul Roychoudhuri^1^, Hosni Hussein^1,9^^, David Klenerman^5^, Kourosh Saeb-Parsy^2^ and Bidesh Mahata^1*^

Correspondence to: [bm562@cam.ac.uk](mailto:bm562@cam.ac.uk)

**This PDF file includes:**

Figures. S1 to S11

Captions for Data S1 to S11

**Figure. S1.**


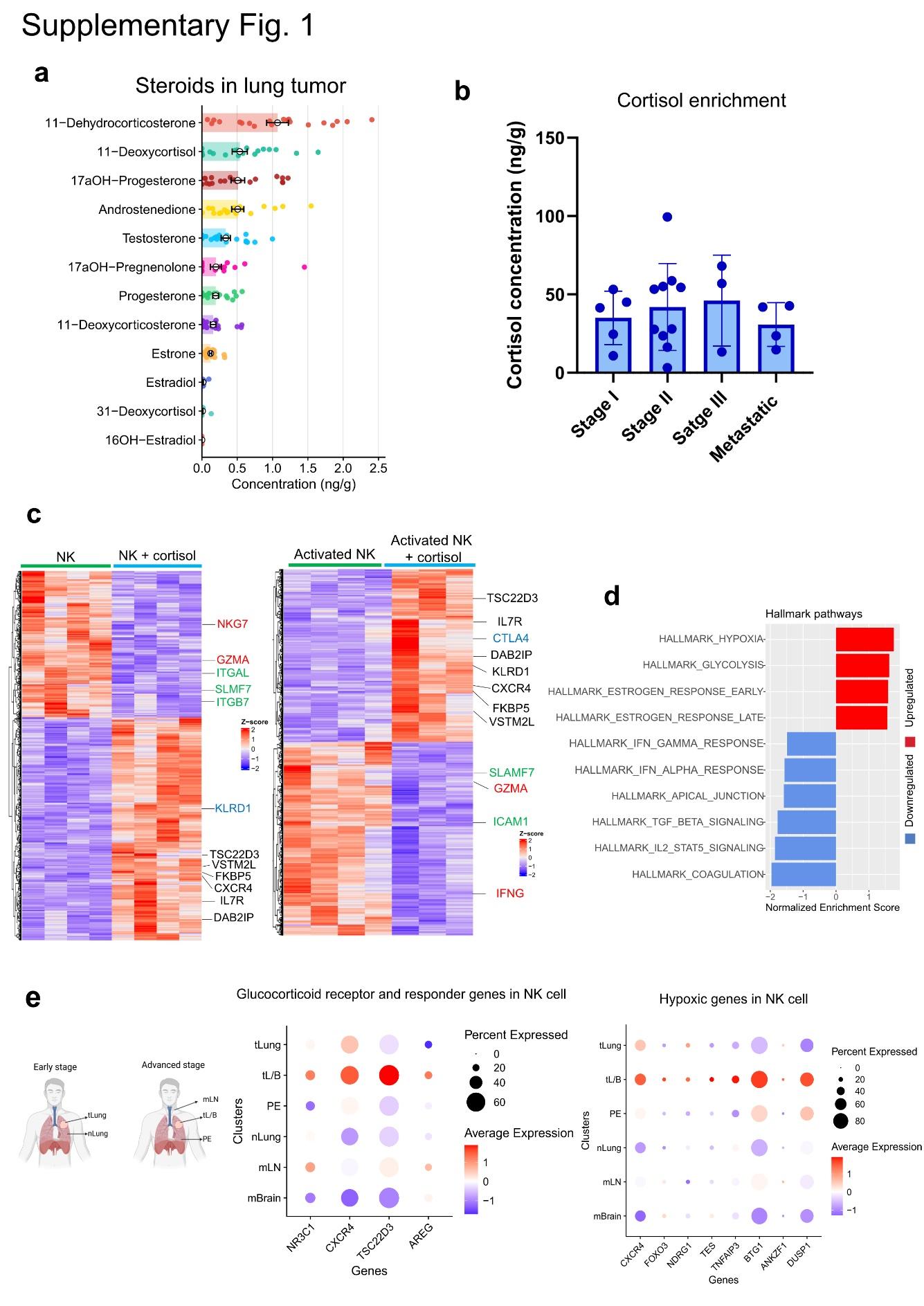


**Supplementary Fig. 1: Steroid abundance, glucocorticoid signaling, and its association with hypoxia across the Lung TME.**

1. The concentration of different steroids (in ng/g of tissue) in lung tumor tissue was estimated using LC-MS/MS. The less abundant steroids (the mean concentration is nearly or less than 1 ng/g) have been shown here. (Data are represented as mean ± SEM, n=20).
2. Cortisol levels in different stages of lung cancer as detected in LC-MS/MS analysis of 22 samples (Data are represented as mean ± SEM, Stage I: n = 5, Stage II: n = 10, Stage III: n = 3, metastatic: n = 4).
3. Heatmaps of transcriptomic changes in resting and activated (cocultured with K562) primary human NK cells following cortisol treatment (1 µM, 6 h). Cortisol exposure downregulated activation-associated transcripts (*NKG7*, *GZMA*) and upregulated glucocorticoid-responsive genes (*TSC22D3, FKBP5, DAB2IP*).
4. Hallmark pathway enrichment analysis showing upregulation of hypoxia and glycolysis signatures in NK cells and suppression of IFN-γ, IL-2/STAT5, and TGF-β signaling upon cortisol treatment.
5. Dot-plots showing expression of glucocorticoid receptor and responsive genes (*NR3C1*, *CXCR4*, *TSC22D3*, *AREG*) and hypoxia-associated genes across NK cells from early- and advanced-stage lung tumors and matched tissues. The left illustration was created using BioRender.

**Figure. S2.**


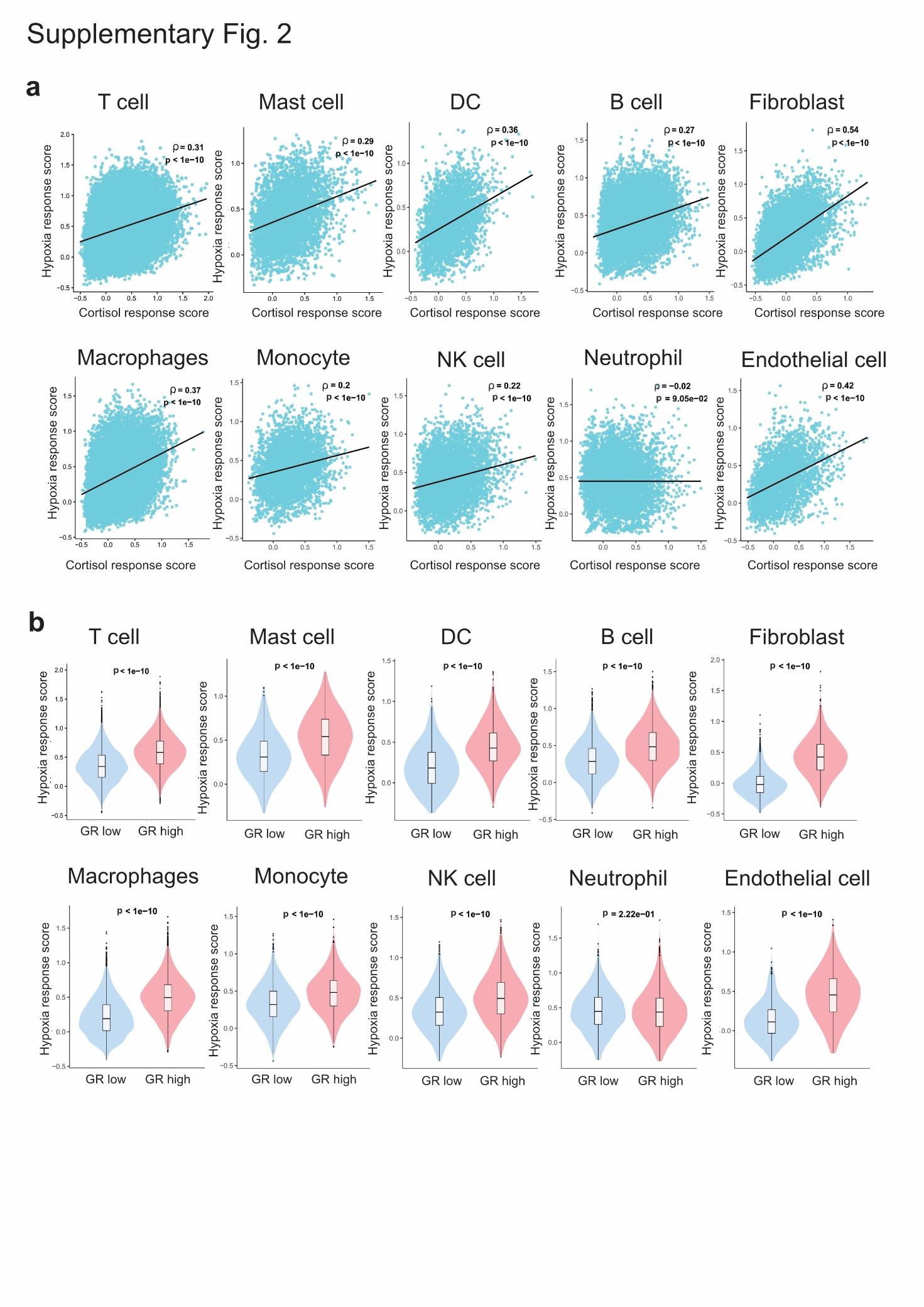


**Supplementary Fig. 2: Glucocorticoid signaling is associated with elevated hypoxia-response signatures across immune and stromal compartments**

1. Scatter plots showing the correlation between cortisol response scores and hypoxia response scores across major tumor-infiltrating cell types, including T cells, mast cells, dendritic cells (DCs), B cells, fibroblasts, macrophages, monocytes, NK cells, neutrophils, and endothelial cells. Each point represents a single cell. Spearman correlation coefficients (ρ) and associated p values are indicated for each population. Cell populations have been collected from scRNA seq of 103 lung cancer patient samples.
2. Violin plots comparing hypoxia response scores between GR_low (lower expression of glucocorticoid receptor) and GR_high (higher expression of glucocorticoid receptor) cells for each immune and stromal subset. GR_high cells consistently display markedly higher hypoxia activity than GR_low counterparts across nearly all cell lineages except neutrophil (p < 1×10⁻¹⁰ for all comparisons except neutrophil, Wilcoxon rank-sum test). Cell populations have been collected from scRNA seq of 103 lung cancer patient samples.

**Figure. S3.**

**
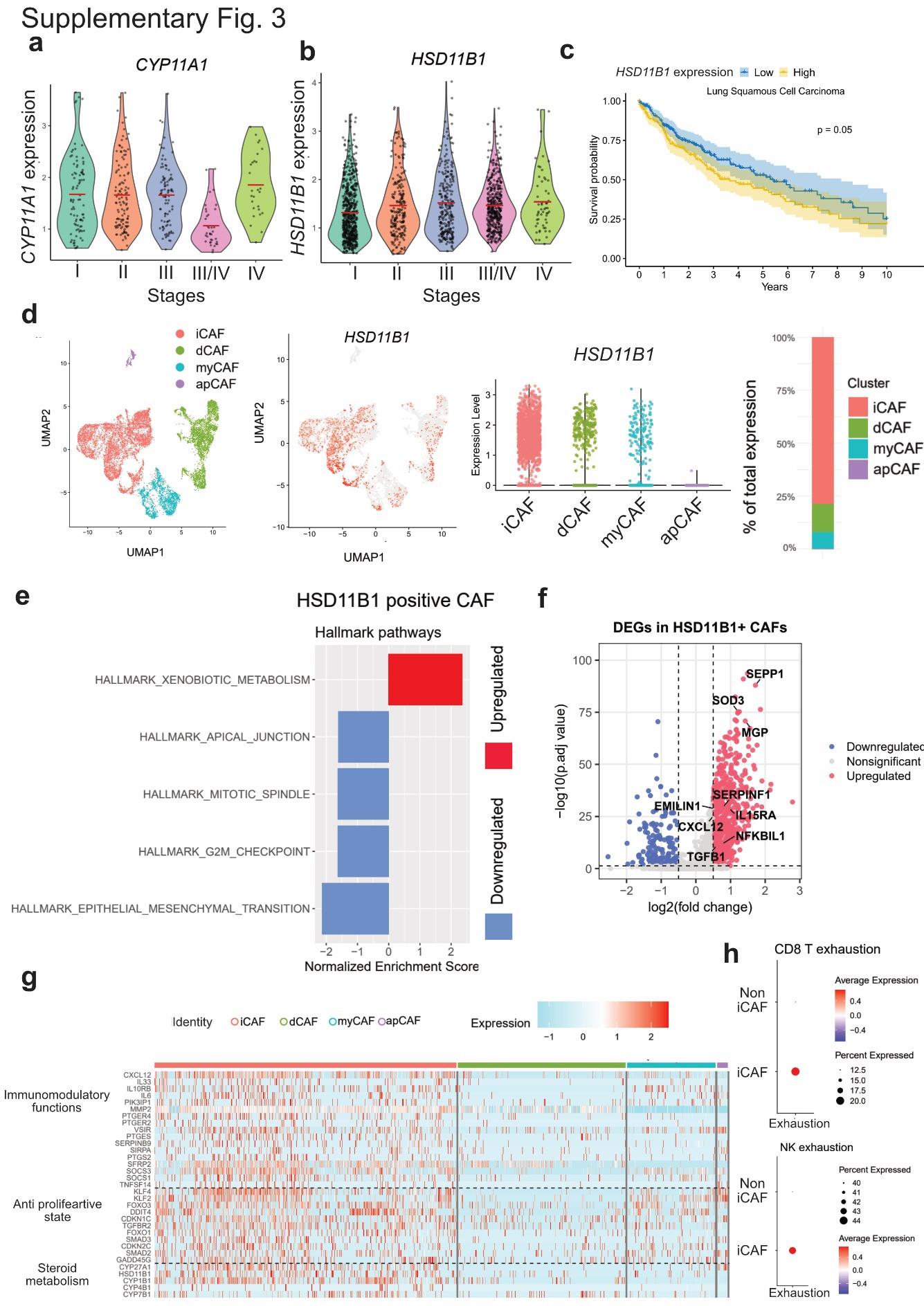
**

**Supplementary Fig. 3: Cortisol recycling CAFs and macrophages influence anti-tumor immunity in lung TME**

1. Violin plots showing stage-wise expression of *CYP11A1* in single-cell RNA seq data from 103 lung cancer patients.
2. Violin plots showing stage-wise expression of *HSD11B1* in single-cell RNA seq data from 103 lung cancer patients.
3. Kaplan–Meier survival analysis of LUSC patients stratified by *HSD11B1* expression (high vs. low, median split). High *HSD11B1* expression was associated with a trend of reduced overall survival (p = 0.05, log-rank test).
4. UMAP visualization and violin plots showing *HSD11B1* expression among fibroblast subtypes, namely, inflammatory CAFs (iCAFs), myofibroblastic CAFs (myCAFs), antigen-presenting CAFs (apCAFs), and desmoplastic CAFs (dCAFs). *HSD11B1* was most strongly expressed in iCAFs.
5. Hallmark pathways significantly enriched in HSD11B1^+^ CAFs compared with HSD11B1^-^ CAFs. Positive NES values indicate upregulated pathways (red), whereas negative NES values indicate downregulated pathways (blue).
6. Volcano plot showing differentially expressed genes in HSD11B1^+^ CAFs.
7. Heatmap of chemokines, cytokines, and steroid-metabolizing genes across fibroblast subtypes (iCAF, dCAF, myCAF, apCAF). iCAFs were enriched for immunomodulatory factors (CXCL1, IL6, CCL2, PTGS2) and cortisol-recycling enzymes (HSD11B1, CYP1B1), supporting their role as key paracrine mediators of inflammation and local steroid regeneration within the tumor microenvironment**.**
8. Dot-plot representation of CD8^+^ T-cell exhaustion (top) and NK-cell exhaustion (bottom) signatures in iCAF versus non-iCAF populations. Dot size reflects the percentage of cells expressing the exhaustion genes, and dot color indicates the average expression level. iCAF-rich samples show a stronger immunosuppressive phenotype.

**Figure. S4.**


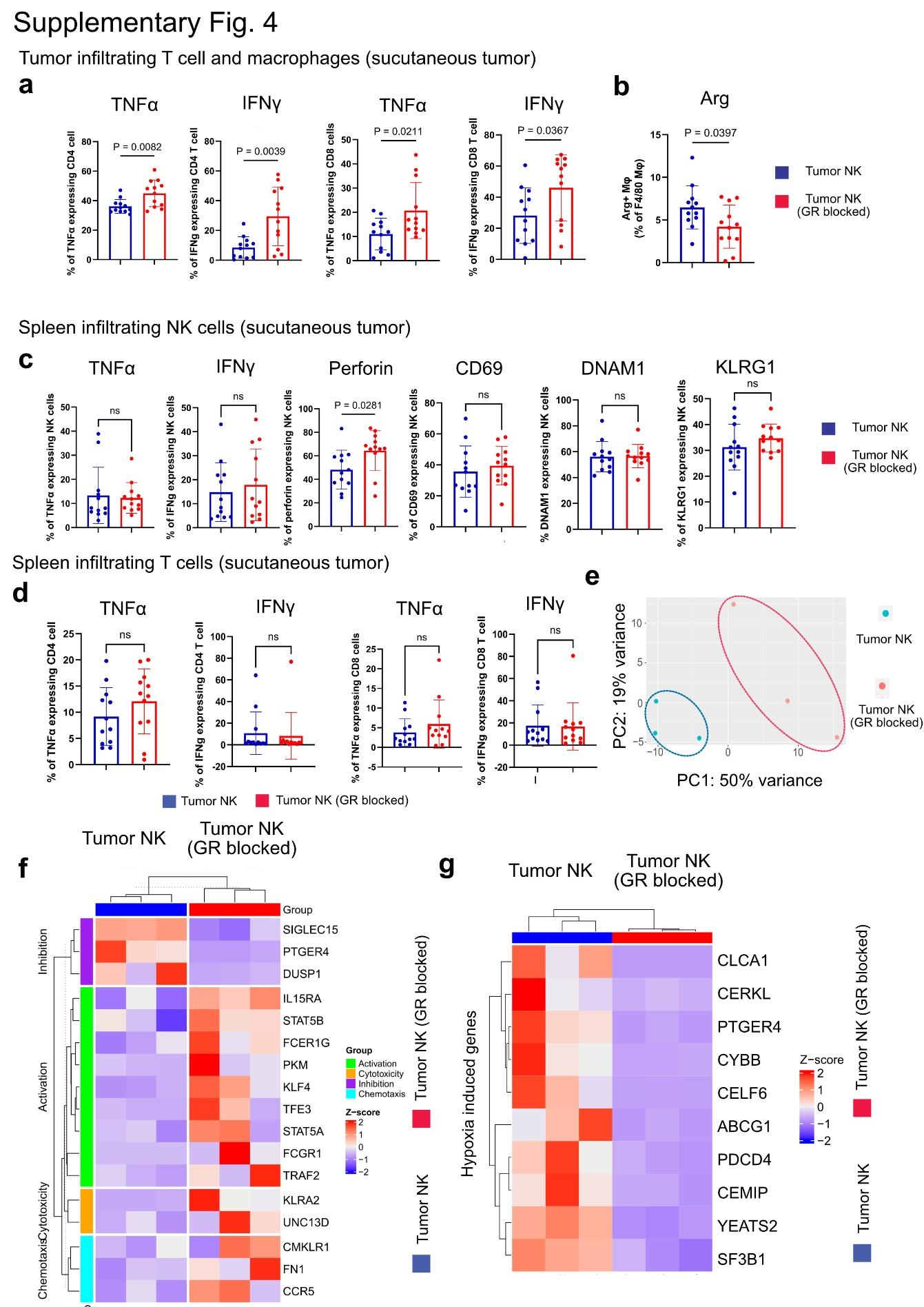


**Supplementary Fig. 4: GR blockade enhances NK-cell effector function in lung tumors in mice.**

1. Flow cytometric quantification of T cells from subcutaneous LLC-OVA tumors showing a significant increase of TNFα⁺ and IFNγ⁺ in tumor-infiltrating CD4⁺ and CD8⁺ T cells following GR (Glucocorticoid receptor) blockade. (Data are represented as mean ± SEM, n = 12; unpaired two-tailed t-test).
2. Flow cytometry analysis of tumor-associated macrophages from the same cohort showing reduced arginase-1 (Arg1) expression upon GR blockade, suggesting partial repolarization from an immunosuppressive to an inflammatory phenotype. (Data are represented as mean ± SEM, n = 12; unpaired two-tailed t-test).
3. Flow cytometry analysis of spleen-infiltrating NK cells from subcutaneous tumor-bearing mice showing no significant changes in major activation and cytotoxic markers of NK cells, indicating limited systemic effects of GR inhibition on peripheral NK populations. (Data are represented as mean ± SEM, n = 12; unpaired two-tailed t-test). Gating: All cell>Singlets>Live cell>CD45^+^>CD3^-^CD19^-^>NK1.1^+^
4. Flow cytometry analysis of spleen-infiltrating CD4⁺ and CD8⁺ T cells showing no significant difference in TNFα or IFNγ-producing CD4⁺ and CD8⁺ T cells between vehicle- and GR-blocked groups. (Data are represented as mean ± SEM, n = 12; unpaired two-tailed t-test). Gating: All cell>Singlets>Live cell>CD45^+^>CD4^+^TCRb^+^ or CD8^+^TCRb^+^
5. Principal component analysis (PCA) of tumor-infiltrating NK cell transcriptomes demonstrating distinct clustering between GR-blocked and vehicle-treated groups.
6. Heatmap showing key transcriptional changes in tumor-infiltrating NK cells after GR blockade. GR inhibition upregulated genes associated with activation, cytotoxicity, and chemotaxis while downregulating inhibitory genes.
7. Heatmap depicting repression of hypoxia-induced stress-response genes in GR-blocked NK cells, highlighting restoration of metabolic and effector homeostasis within the tumor microenvironment.

**Figure. S5.**


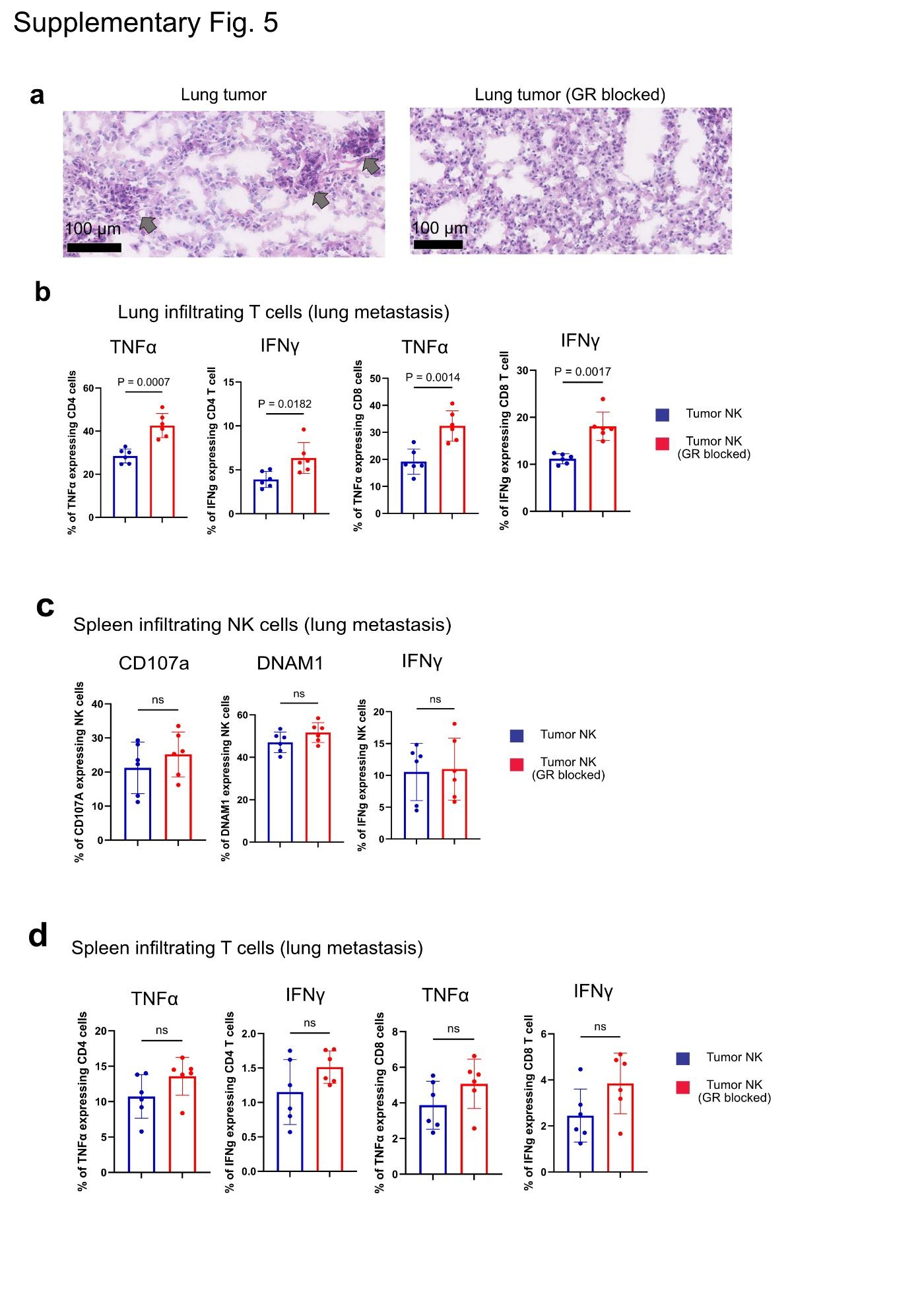


**Supplementary Fig. 5: GR blockade reduces lung tumor burden and increases anti-tumor immune response.**

**a.** Representative histological images of lung metastases from mice treated with vehicle or GR-blocker showing reduced tumor burden and restoration of alveolar architecture in the GR-blocked group. Scale bar, 100 µm.

**b.** Flow cytometric quantification of lung-infiltrating T cells in the metastasis model showing significantly higher TNFα⁺ and IFNγ⁺ CD4⁺ and CD8⁺ T cells in GR-blocked mice. (Data are represented as mean ± SEM, n = 6; unpaired two-tailed t-test). Gating: All cell>Singlets>Live cell>CD45^+^>CD4^+^TCRb^+^ or CD8^+^TCRb^+^

**c.** Flow cytometry analysis of spleen-infiltrating NK cells from the lung metastasis model showing comparable CD107a, DNAM1, and IFNγ expression between control and GR-blocked groups, suggesting local (tumor-restricted) rather than systemic immunomodulation. (Data are represented as mean ± SEM, n = 6; unpaired two-tailed t-test). Gating: All cell>Singlets>Live cell>CD45^+^>CD3^-^CD19^-^>NK1.1^+^

**d.** Flow cytometry quantification of spleen-infiltrating CD4⁺ and CD8⁺ T cells in the lung metastasis model showing no significant differences in TNFα⁺ and IFNγ⁺ CD4⁺ and CD8⁺ T cells in GR-blocked mice. (Data are represented as mean ± SEM, n = 6; unpaired two-tailed t-test). Gating: All cell>Singlets>Live cell>CD45^+^>CD4^+^TCRb^+^ or CD8^+^TCRb^+^

**Figure. S6.**


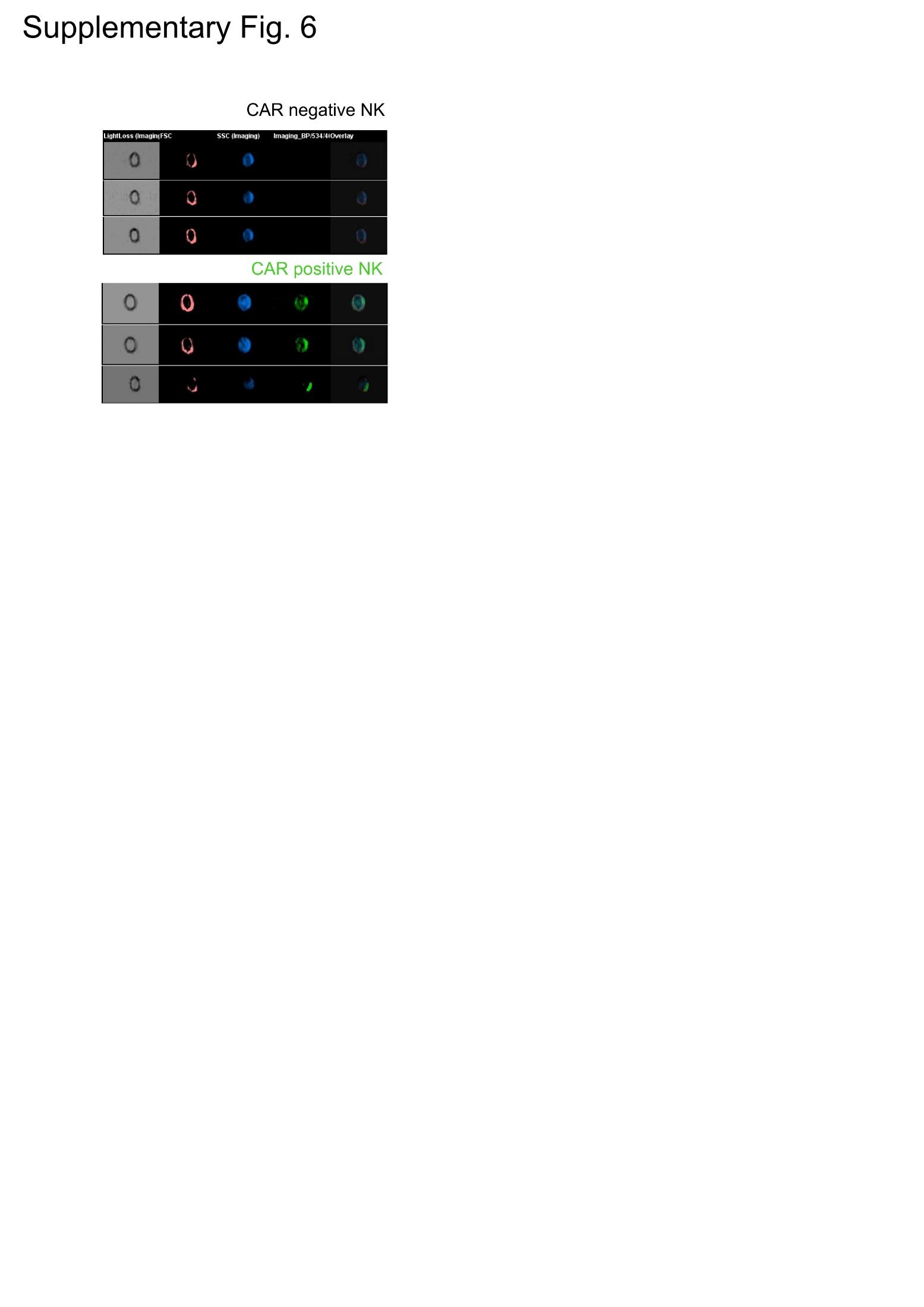


**Supplementary Fig. 6: CEACAM5-specific CAR-NKs target CEACAM5-expressing cells.**

Representative imaging cytometry (BD FACSDiscover™ S8 Cell Sorter with BD CellView) analysis of CEACAM5-CAR expression in transfected NK cells. CAR-negative NK cells showed no detectable signal, whereas CAR-positive NK cells exhibited strong membrane-localized fluorescence, confirming efficient CAR expression and surface trafficking.

**Figure. S7.**


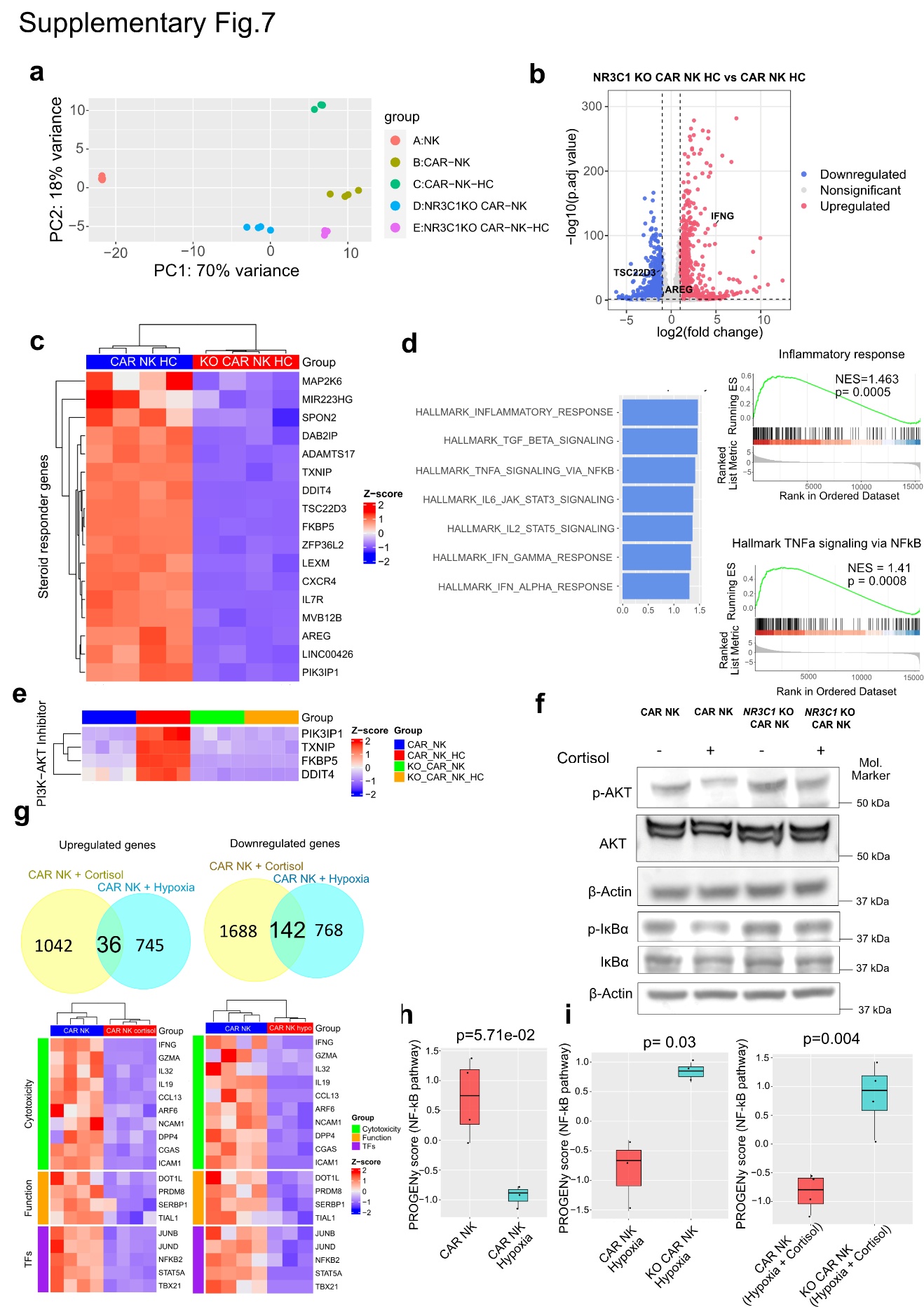


**Supplementary Fig. 7: Glucocorticoid-resistant CEACAM5-specific CAR-NK cells retain tumor-killing activity despite glucocorticoid exposure.**

1. Principal component analysis (PCA) of RNA-seq data showing distinct clustering of CAR-NK, CAR-NK + hydrocortisone (HC, also known as cortisol), and *NR3C1*-KO CAR-NK + HC groups.
2. Volcano plot of differentially expressed genes between activated *NR3C1*-KO CAR-NK and CAR-NK cells in the presence of hydrocortisone. (n= 4 in each group).
3. Heatmap depicting expression of canonical glucocorticoid-responsive genes. Cortisol markedly upregulated *AREG,* *FKBP5*, *TSC22D3*, *DAB2IP*, and *CXCR4* in CAR-NK cells, whereas these genes were suppressed in *NR3C1*-KO CAR-NK cells, confirming functional ablation of glucocorticoid signaling.
4. Gene-set enrichment analysis showing enrichment of Hallmark Inflammatory Response and Hallmark TNFα signaling via NF-κB pathways in *NR3C1*-KO CAR-NK cells compared with cortisol-treated CAR-NK cells, indicating restoration of pro-inflammatory transcriptional activity.
5. Heatmap of PI3K–AKT pathway inhibitors (*PIK3IP1*, *TXNIP*, *FKBP5*, *DDIT4*) showing strong cortisol-induced upregulation in activated CAR-NK cells, which was reversed in activated *NR3C1*-KO CAR-NK cells.
6. Western blot analysis of phosphorylated AKT (p-AKT) and phosphorylated IκBα (p-IκBα) in activated CAR-NK and *NR3C1*-KO CAR-NK cells with or without hydrocortisone treatment. Cortisol suppressed AKT and IκBα phosphorylation in CAR-NK cells but not in *NR3C1*-KO CAR-NK cells, confirming that GR (glucocorticoid receptor) deletion prevents cortisol-mediated inhibition of PI3K–AKT–NF-κB signaling. The nearest molecular ladder (marker) position is shown.
7. Venn diagram showing the number of common genes influenced by cortisol treatment and hypoxic conditions in CAR-NK cells. Heatmaps showing expression of cytotoxicity-related genes (*IFNG*, *GZMA*, *IL32*) and transcription factors (*STAT5A*, *NFκB2*, *TBX21*) across CAR-NK in hypoxic conditions (left) and CAR-NK in cortisol-treated conditions (right).
8. Quantification of PROGENy pathway activity scores showing reduced trend of NF-κB signaling activity in CAR-NK cells under hypoxia (Wilcoxon test, p = 5.71 × 10⁻²).
9. Boxplots of PROGENy-derived NF-κB pathway scores activated CAR NK and *NR3C1*-KO CAR-NK cells under hypoxia ± cortisol conditions, demonstrating recovery of NF-κB pathways in *NR3C1*-KO CAR-NK cells (p *< 0.05*, Wilcoxon test).

**Figure S8.**

**
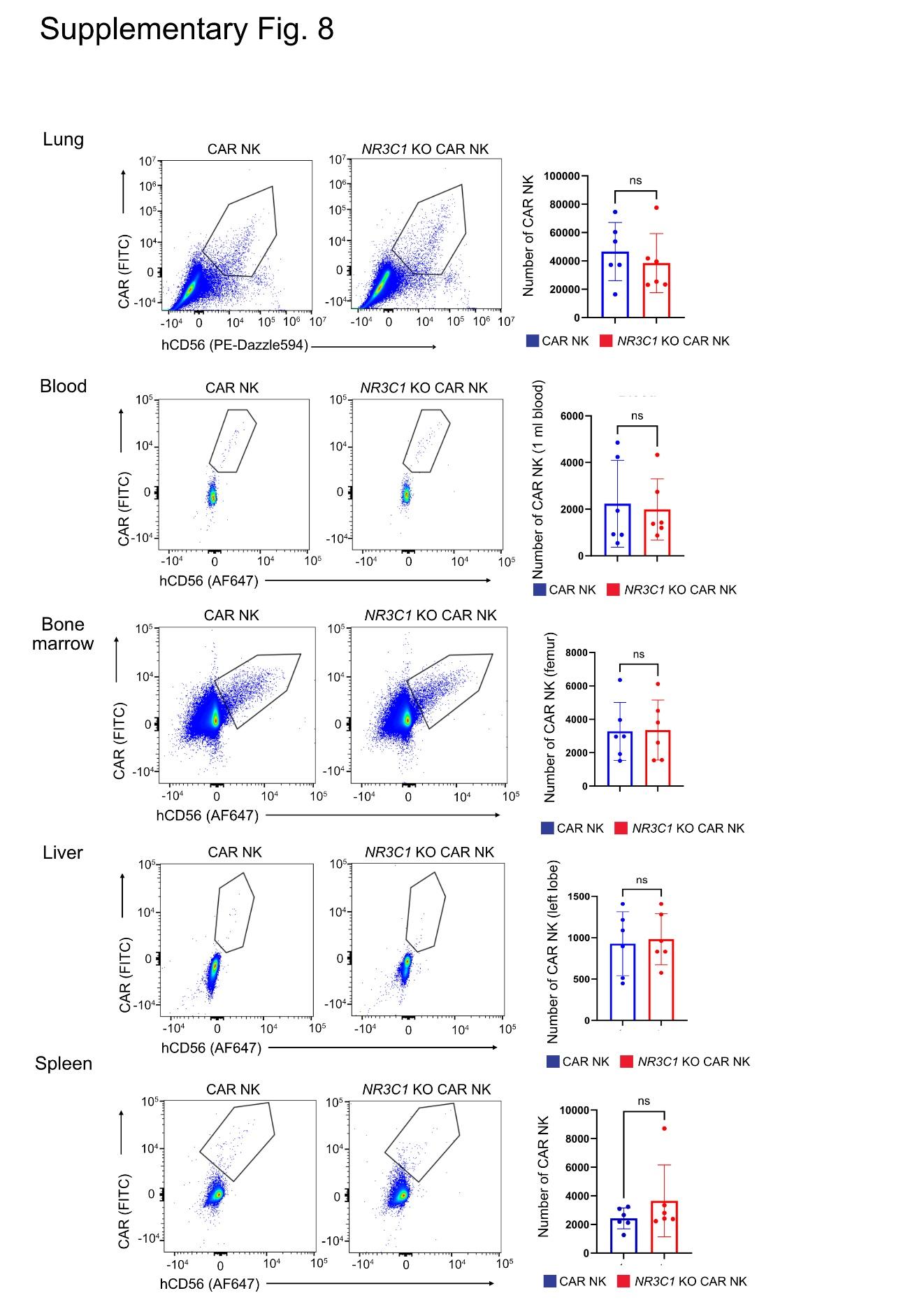
**

**Supplementary Fig. 8: Tissue distribution of CAR-NK and *NR3C1-*KO CAR-NK**

Quantification of human CAR-NK and *NR3C1-*KO CAR-NK cells isolated from lung, blood, bone marrow, liver, and spleen of tumor-bearing mice 14 days post-adoptive transfer. Both groups showed comparable systemic engraftment, indicating that GR deletion does not alter in vivo trafficking or persistence of CAR-NK cells (Data are represented as mean ± SEM, n = 6 per group; unpaired two-tailed t-test, ns = not significant).

**Figure. S9.**

**
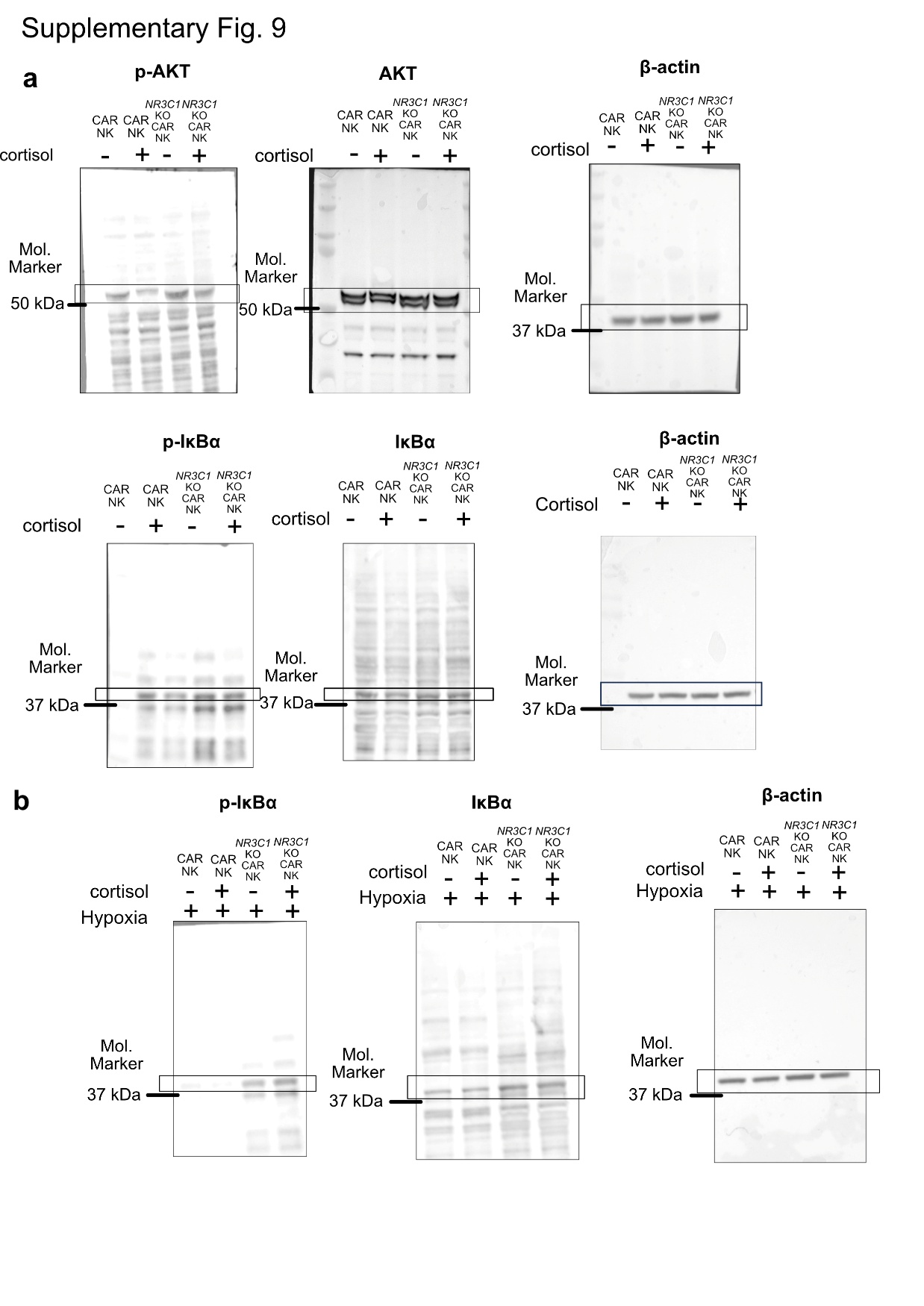
**

**Supplementary Fig. 9: Original western blots**

**a.** Original western blot corresponding to supplementary Fig. 7f. The nearest molecular ladder (molecular marker) position is shown.

**b.** Original western blot corresponding to Fig. 5h. The nearest molecular ladder (molecular marker) position is shown.

**Figure. S10.**


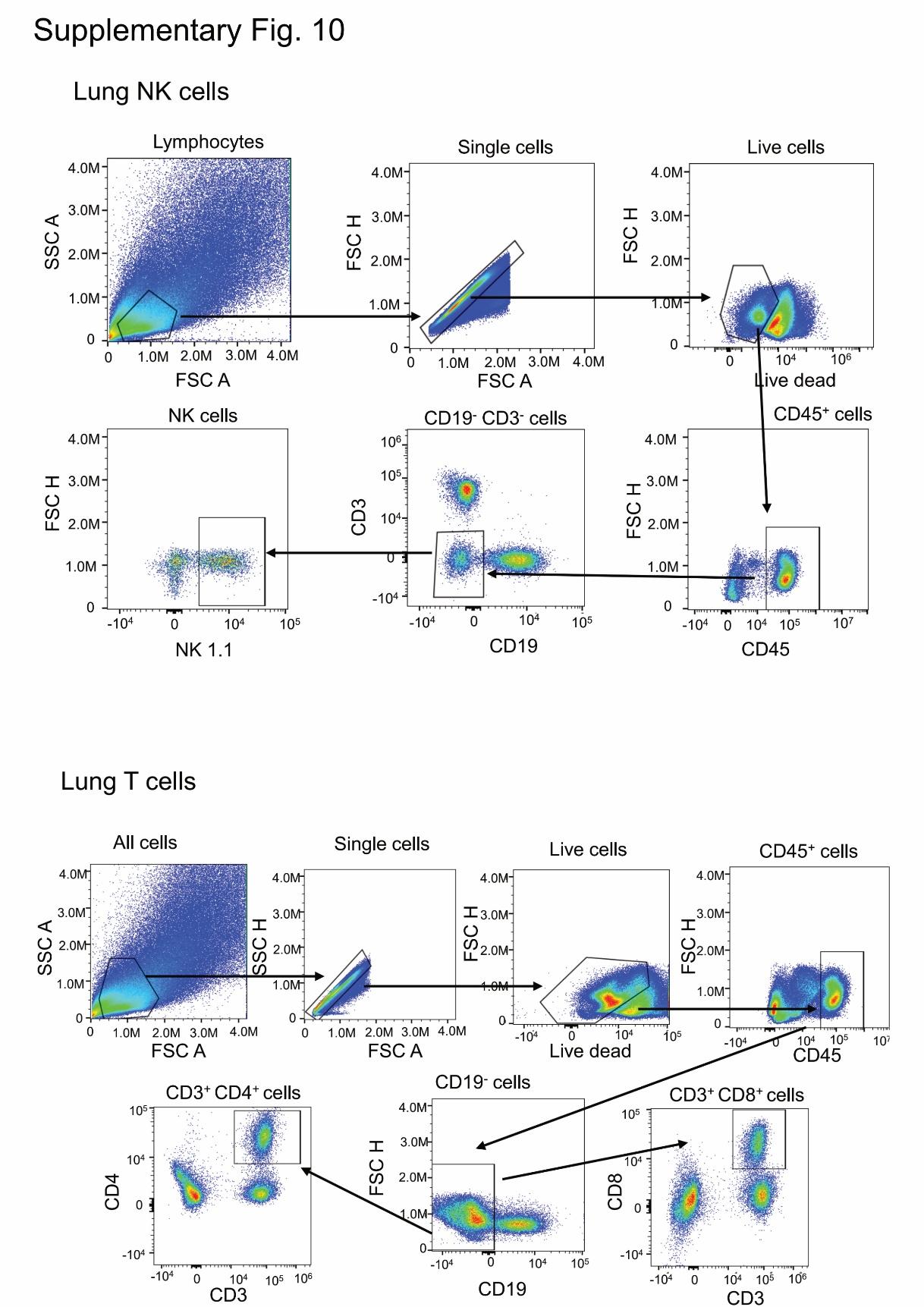


**Supplementary Fig. 10: FACS gating strategies**

Representative flow cytometry gating schemes used to define immune cell populations in lung.

**Figure. S11.**


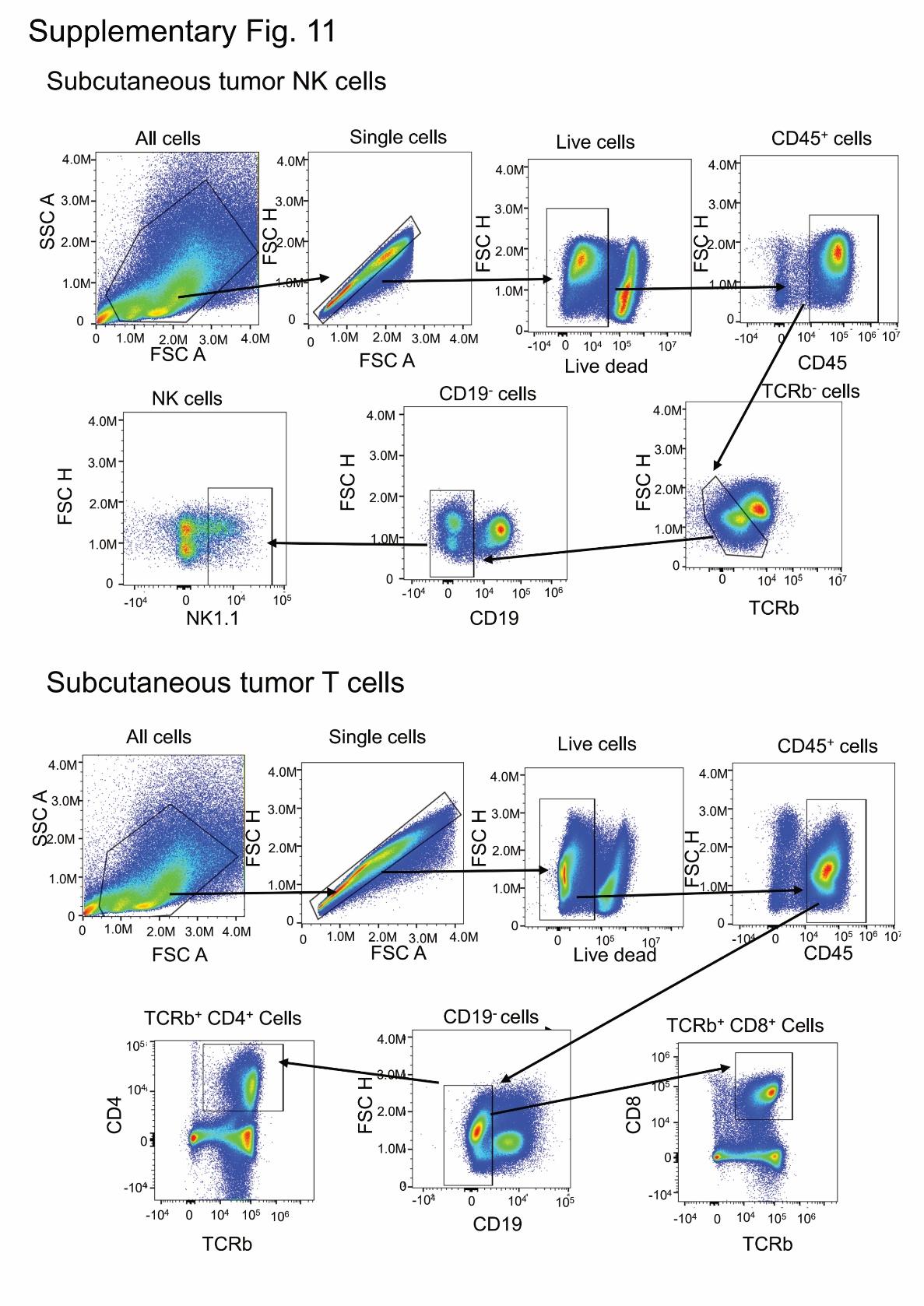


**Supplementary Fig. 11: FACS gating strategies**

Representative flow cytometry gating schemes used to define immune cell populations in the subcutaneous tumor.
